# High Affinity vs. Native Fibronectin in the Modulation of αvβ3 Integrin Conformational Dynamics: Insights from Computational Analyses and Implications for Molecular Design

**DOI:** 10.1371/journal.pcbi.1005334

**Published:** 2017-01-23

**Authors:** Antonella Paladino, Monica Civera, Laura Belvisi, Giorgio Colombo

**Affiliations:** 1 Istituto di Chimica del Riconoscimento Molecolare CNR, Milan, Italy; 2 Dipartimento di Chimica, Università degli Studi di Milano, Milan, Italy; Fudan University, CHINA

## Abstract

Understanding how binding events modulate functional motions of multidomain proteins is a major issue in chemical biology. We address several aspects of this problem by analyzing the differential dynamics of αvβ3 integrin bound to wild type (wtFN10, agonist) or high affinity (hFN10, antagonist) mutants of fibronectin. We compare the dynamics of complexes from large-scale domain motions to inter-residue coordinated fluctuations to characterize the distinctive traits of conformational evolution and shed light on the determinants of differential αvβ3 activation induced by different FN sequences. We propose an allosteric model for ligand-based integrin modulation: the conserved integrin binding pocket anchors the ligand, while different residues on the two FN10’s act as the drivers that reorganize relevant interaction networks, guiding the shift towards inactive (hFN10-bound) or active states (wtFN10-bound). We discuss the implications of results for the design of integrin inhibitors.

## Author Summary

Interactions between proteins are at the basis of all biological processes in the cell. In this context, the study of conformational responses of protein receptors to the binding of endogenous ligands may be a source of inspiration for the design of small molecule modulators that permit to control such biological processes. To progress towards this goal, we need to unravel the molecular determinants that underlie the correlations between sequences, structures and the onset of functional motions in the receptor, eventually illuminating the links between the fine atomic-scale protein-ligand interactions and the large-scale protein motions. Herein, we have concentrated on the multidomain receptor αvβ3 integrin bound to two different sequences of the endogenous ligand fibronectin: the wild type one, wtFN10, which acts as an agonist activating the receptor, and a high affinity mutant, hFN10, which acts as a true antagonist inhibiting the receptor. Through the comparative analysis of several dynamic descriptors at different levels of resolution, from the residue to domain level, we shed light on the salient conformational dynamics differences determined by fibronectin sequence mutations: we show that it is possible to identify interaction hotspots in the integrin binding site that specifically respond to the fibronectin sequence variations, and allosterically drive conformational changes towards integrin activation (in the case of wtFN10 binding) or inhibition (hFN10 binding). Finally we propose an allosteric model of integrin regulation that can be used in the design of small molecule integrin inhibitors or modulators.

## Introduction

Integrins are heterodimeric cell adhesion receptors, composed by the association of α and β subunits. They are typically characterized by a bilobular head and two legs that span the plasma membrane. Integrin ectodomains have been crystallized in a bent, genuflexed conformation (corresponding to the inactive or closed state) as well as in an open one (corresponding to the active state) with high-affinity for ligands. [1,2] Conformational changes from the bent to the open structures in integrin extracellular, transmembrane and cytoplasmic domains underlie a diverse range of biological processes, including cell migration, morphogenesis, immune responses, vascular haemostasis, cell-to-cell interaction and intracellular signal transduction. The dysregulation of these processes contributes to the pathogenesis of many diseases. [3] In particular, αvβ3, αvβ5 and α5β1 integrins are involved in angiogenesis, tumor progression and metastasis, whereas the platelet αIIbβ3 receptor is central to haemostasis and contributes to thrombosis. [4–7]

Determination of the crystal structure of the ectodomain of αvβ3 in the absence and presence of a prototypical RGD ligand unveiled the modular nature of integrins and clarified the basis of the divalent cation—mediated interaction with extracellular ligands. [1,8]

**Fig 1:**
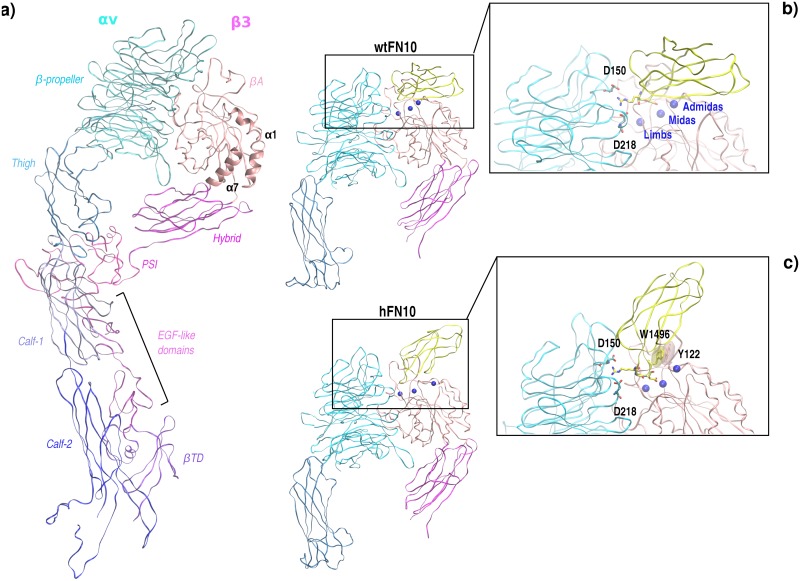
3D-structure of integrin αvβ3 and αvβ3-FN10 complexes. a) Full-length αvβ3 ectodomain representation. αv domains are labeled and colored with shades of blue, β3 domains are shown with shades of pink. For clarity, αvβ3 is displayed in the extended conformation. α1 and α7 helices of the β3 chain are labeled and shown in cartoon. b-c) αvβ3-wtFN10 and αvβ3-htFN10 complexes. Cyan and pink color codes are used for αv and β3 subunits, respectively. Fibronectin domain is displayed in yellow ribbons, blue spheres represent Mn^2+^ ions at metal-binding sites. Insets: blowup of the FN-RGD motif and Mn^2+^ at metal-binding sites. D150_αv_ and D218_αv_ of the electrostatic clamp are labeled. In c) W1496_FN_ and Y122_β3_ are indicated with ghost surfaces. See main text.

The ectodomain of αvβ3 revealed a “head” attached to two “legs” in the native, full-length integrin, whereby the legs connect to short transmembrane and cytoplasmic segments. The integrin head consists of the seven-bladed β-propeller domain from the αV non-covalently bound to the βA domain of the β3 subunit (Fig 1a). The αV leg is formed by an Ig-like “thigh” domain attached to two large colinear β-sandwich domains, designated calf-1 and calf-2. The β3 leg is formed by an Ig-like hybrid domain, the βA projecting from one of its loops, a PSI domain, four EGF-like domains, and a membrane proximal β-tail domain (βTD). The RGD sequence of physiologic ligands typically engages αvβ3 in a cleft between the β-propeller and βA domains, producing a characteristic electrostatic clamp between the guanidinium moiety of the RGD-triad and aspartic acids of the αv subunit and, at the same time, enabling RGD-Asp(Oδ1/Oδ2) to coordinate a metal ion at MIDAS (Metal Ion-Dependent Adhesion Site) of the β3 subunit (Fig 1b and 1c). [9]

The crystal structures of the bound and unbound forms of αIIbβ3 and αvβ3 provide a structural model of the ligand-effects on the two proteins, and of their activation mechanism. [2,8,10] In this context, experiments have shown that ligand binding to the head of αIIbβ3 induces the opening of the hinge between the βA and hybrid headpiece domains, implying a transition from the closed unbound state to the open one upon ligand binding. [2,11–14] Different structural observations, based on soaking the RGD containing drug cilengitide into αvβ3 crystals have implied that the hinge is closed even in the presence of the ligand. [1,15]

According to structural modeling based on EM-data for αvβ3, and on EM, SAXS cryo-tomography and FRET for αIIbβ3, extension at the knees unclasps integrins from a compact bent conformation, where the legs are bent at the knees and folded back against the head. Subsequently, in integrin headpiece opening towards the active state, the hybrid domain swings out, the βA domain evolves to the open conformation, and affinity for other endogenous ligands increases. The bend occurs between the thigh and calf-1 domain of αV and between EGF domain 1 and 2 of β3. [8,11] Adair and co-workers suggested that ligand binding provides the energy for additional conformational changes, including perhaps genu-extension, thus triggering integrin out-in activation and signaling. [16]

The conformational changes vary with cell type and the state and nature of the ligand. [8,11,17]

Endogenous integrin-ligands include proteins such as fibronectin, fibrinogen, vitronectin, collagen, laminin, that mediate the coupling between the cell and the extracellular matrix (ECM) and cellular cytoskeleton through adaptor molecules like actin, talin, filamin etc. [17–20]

The chemical and structural properties of the framework around the RGD sequence and the complementary features displayed by the integrin binding pockets have been shown to affect recognition between the partners and to determine the functional consequences of the interaction. [3]

Fibronectin (FN) is a prominent example of how differential recognition between sequences is translated into different functional consequences. FN is a widely expressed ECM protein and a promiscuous ligand for integrins as well as for numerous other cell adhesion receptors. FN exists as a soluble dimeric glycoprotein of two monomers, each of them composed by three repeating modules.

As in the case of other integrin ligands, FN-integrin interactions are mediated by the RGD motif located on the 10th type III repeat.

Recently the 3D structures of αvβ3 bound to the 10th type III RGD domain of wild-type fibronectin (wtFN10) and its high affinity mutant (hFN10) have been solved by Arnaout and collaborators (Fig 1b and 1c). [3] The main differences between the two mutants are found in the sequences flanking the RGD binding motif: specifically, wild-type fibronectin (wtFN10) sequence -G**RGD**SPAS- is replaced by -P**RGD**WNEG- in high affinity fibronectin (hFN10).

hFN10 -P**RGD**WNEG- sequence is more polar compared to wild-type -G**RGD**SPAS-; it also displays a larger surface due to the presence of the tryptophan substitution. These sequence differences appear to modulate the ligand effects on the integrin, ultimately affecting integrin activation. Indeed, while wtFN10 represents an integrin agonist, hFN10 behaves as a real antagonist. [3]

Crystal structures of the αvβ3–hFN10 complex (pdb code: 4MMZ) provided an important structural framework to investigate the activity of hFN10 as a pure antagonist; here, the novel W1496(hFN)-Y122(αvβ3) π-π interaction is hypothesized to ‘freeze’ the integrin in an inactive conformation (Fig 1c). This βA tyrosine is largely conserved also in α5β1 and β2 integrins, supporting its key function in the activation process. [3] Moreover, the inward movement of Y122-βA described for the wild type FN (wtFN10) complex (pdb code: 4MMX) would be incompatible with the integrin-hFN10 crystal structure where Y122 aromatic ring would clash with mutated fibronectin residue W1496_hFN_.

From biophysical data and cell-based assays, hFN10 actually behaves as a pure antagonist, which does not induce activation-specific LIBS (Ligand Induced Binding Site epitopes) expression and also reduces cell spreading, that is an index of outside-in signaling by ligand-occupied integrins. Moreover, it does not affect the hydrodynamic radius of the soluble αvβ3 ectodomain, indicating a stable compact/bent form. [3]

The above-mentioned studies and the availability of high resolution crystal structures of the two complexes provide an optimal starting point to investigate the differential aspects of functional dynamics induced by limited sequence differences in the FN ligands when bound to αvβ3, and consequently shed light onto the determinants of integrin activation processes.

Herein, we set out to compare several aspects of the dynamics of integrin αvβ3 in complex with wtFN10 or hFN10, as well as in the unbound state (apo), that can be linked to observed biological activities of the molecules.

To progress along this route, we carry out microsecond long MD simulations of the two complexes and compare their dynamic evolution from the fine level of inter-residue coordinated fluctuations to larger scale domain motions. We characterize the main distinctive traits of conformational evolution of the two complexes and propose a model for the determinants of FN-induced differential activation of αvβ3. It is to be noted that the main differences between the two complexes emerge at the level of internal dynamics and local reorganizations (the RMSD between the X-ray structures of the integrin in the wtFN10 and hFN10 complexes is limited to 0.2 nm). Indeed, while no major spontaneous transition from the analogous starting crystal structures can be reported, the local structural and chemical organization similarity of the β3 subunit and of the coordination at the MIDAS/ADMIDAS sites between the wtFN10 complex and the S7/S8 states observed by Springer and coworkers [10] for α_IIb_β_3_ may be indicative of the higher tendency for the wt complex to favor transition to the open state. Finally, we discuss the implications of our results for the design and optimization of ligand-mimetic integrin inhibitors.

## Results

In this work, MD studies of αvβ3 in complex with the two forms of fibronectin, *wild type* (wtFN10) and mutated high affinity (hFN10), are started from the two recently published crystal structures (4MMX, 4MMZ). [3] Three replicas of 500 ns (3*0.5 μs = 1.5 μs) of all-atom simulation are run for each system. Analogous simulations are run for the unbound integrin as a control. We performed unbiased MD simulations on the upper ectodomain, by cutting α and β chains at the Thigh and Hybrid domains, respectively (see methods), in line with previous studies to investigate intra- and interdomain interactions responsible for ligand-induced integrin conformational dynamics. [21–24] As a general control, to estimate the extent of potential artifacts due to using truncated domains and limited length scales, we carried out GNM analysis [25–26] on the full-length as well as on the truncated models of complexed integrins. The cumulative contributions of the first two slowest modes to the overall mobility of the protein, and the contributions of the Thigh and Hybrid domains to such slow modes, obtained for the full length X-ray structure and truncated models used for MD simulations are comparable. In particular, a collectivity degree of 0.85 and 0.87 on average for full-length and truncated structures, respectively, gives the extent to which structural elements move together in that particular model. Importantly, the contribution of the thigh domain to the principal eigenvectors is comparable in both systems: as a consequence, despite the presence of a large number of additional contacts/constraints in the full-length structure, the thigh domain undergoes displacements similar to those observed in the truncated model. This lends further support to our initial assumption.

As a general caveat, it must be noted that the simulated time scale, while extensive considering the large dimensions of the systems under study, may still be shorter than the time scale accessible in experiments: in this framework, our goal is not to profile the full conformational changes of the complexes but to illuminate the microscopic details of integrin internal dynamics induced by specific ligands that can be linked to the activation of specific functional states. Previous research demonstrated that even small differences in the structural dynamics at a functional site can be sufficient to set the stage for a modulation of the populations of different functionally oriented states (see also work by Nussinov and coworkers, e.g. refs [27–29]). Here, we show that changing protein flexibility at the local and global levels can lead to the activation/deactivation of dynamic states that can be aptly linked to experimentally probed changes in 3D organization of the integrin.

This is expected to unveil dynamic signatures for different sub-states, providing a basis to model the consequences of binding on the onset of functionally oriented dynamics, as well as providing information for modulator design. Within the limitations of MD sampling, a comparative approach in which computational analysis of the same system in different conditions (the complex and apo states) turns out to be a useful tool to reveal dynamical features that can thus provide information on the salient traits of biologically relevant microscopic motions.

### Structural characterization of αvβ3 in the two different complexes

αvβ3 ectodomain includes β-propeller (aa. 1–438) and thigh (aa. 439–599) domains of subunit α and βA (aa. 109–352) and hybrid (aa. 55–108, 353–434) domains of chain β (Fig 1). Fibronectin subunit extends from aa. 1417 to 1507, engaging the interfaces of αvβ3. The two studied fibronectin molecules target αvβ3 placing the RGD motif at a crevice in the integrin head between the β-propeller and the βA domains making extensive contacts with both. RGD ligands bind to the integrin β subunit via a divalent metal ion located at the top of the βA domain, named the “metal ion-dependent adhesion site” (MIDAS). Two additional ion-binding sites border the βA domain MIDAS on either side, which are termed the “ligand-induced metal-binding site” (LIMBS) and the “adjacent to the MIDAS” (ADMIDAS).

Differences in the sequences near the RGD binding motif at the α/β interface, combined to the different placing of the two FNs relative to the integrin in the complex, may induce dynamic events throughout the integrin structure that can translate into a differential activation of the integrin (Fig 1).

**Fig 2:**
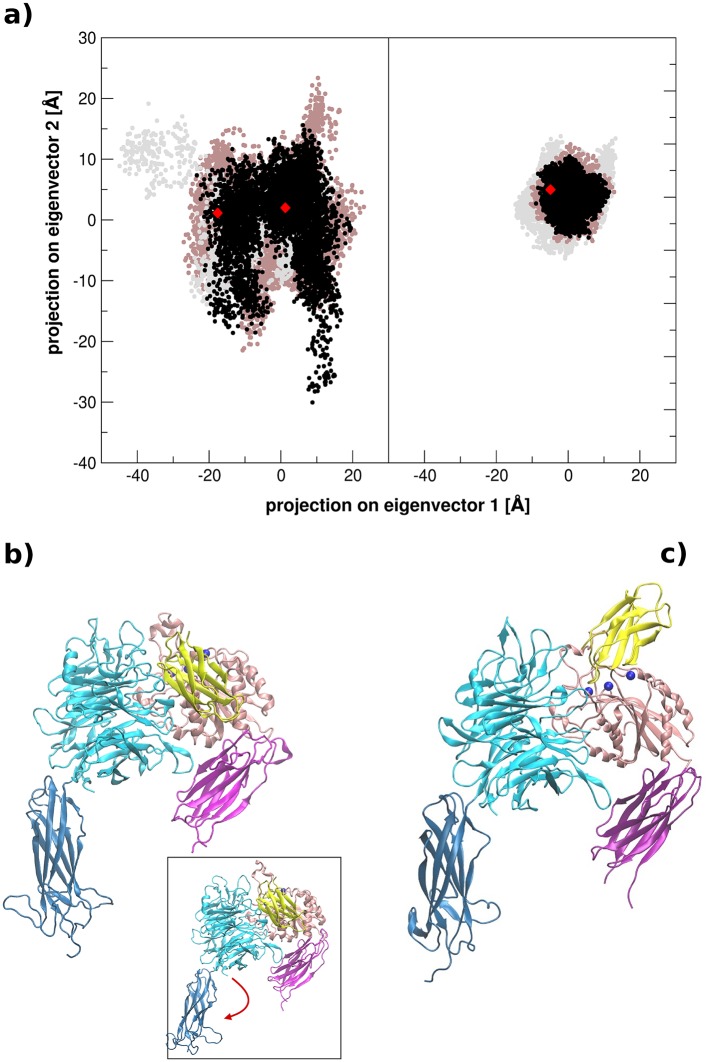
ED analysis. a) Projection of wtFN10 and hFN10 onto the first two principal modes of the simulations (3 replicas). Calculations are carried out on C-alpha atoms of the full-length complex (integrin+FN = 1070 particles). b-c) Representative structures for wtFN10 (b) and hFN10 (c) in replica #1 corresponding to the centroid (red balls) of the populations reported in a). Inset contains wtFN10 of the least populated cluster (left red spot). Red arrow indicates the main structural deviation. Structure representations are given in blue (αv) and pink (β3) colors (integrin) and yellow (fibronectin) cartoons. See also S1 Fig and S1 Table in Supporting Information.

In general, both αvβ3-FN10 complexes are stable and metal coordination, at both β-propeller and integrin-FN interface, is conserved during the whole length of MD simulations. However, significant differences in the structural evolution immediately emerge, indicating the influence of the FN sequences surrounding the RGD motif and of the initial orientations on the dynamics of the complexes. Indeed, Essential Dynamics (ED) [30] analysis (Fig 2a) on the trajectories highlights a rich behavior for the complex with wtFN10, in which two conformational populations are observed, both converging to more compact complex structures than the starting one. In Fig 2b the representative structures of the two clusters are indicated: only a small deviation of the thigh_αv_ domain occurs in the less populated ensemble (Fig 2b inset). In contrast, one main conformational ensemble is populated for the complex with hFN10, which substantially corresponds to the crystallographic structure (Fig 2a and 2c). Conformational variability of the uncomplexed integrin (1JV2) [8] at the level of large-scale displacements is analyzed here as a control by looking at the projections on main eigenvectors from PCA analysis of the respective MD trajectories. It is worth noting here that except for the absence of ligand and of the metal ions (at LIMBS and MIDAS), the X-ray structure of the apo state shows a 0.258 nm RMSD deviation (Calpha atoms) from αvβ3 in wtFN10-bound form (4MMX) and 0.234 nm rms deviation from αvβ3 in hFN10-bound structure (4MMZ). In general the conformational dynamics of the apo state seems to be richer than the one observed for the antagonist bound hFN10-complex, while not sampling all of the conformations of the agonist bound wtFN10-complex. Details of ED analysis are reported in supporting information (S1 and S5 Figs).

The structural transition between integrin active and inactive states is induced by global rearrangements of the headpiece upon fibronectin binding.

To better characterize the global conformational changes that differentiate the two complexes, we analyzed the reciprocal orientations of selected subdomains in αvβ3 and of the bound wtFN10 or hFN10. Accordingly, we defined the principal axes of integrin β3 subunit and fibronectin domains and calculated the correspondent torsion angle along the MD trajectories (see Methods). [31] Consistent with ED findings, fibronectin in the high affinity complex maintains the crystallographic orientation and the torsion angle fluctuates only slightly (+/- 20°), oscillating between 56° and 82°. On the other hand, well-defined rotational motions characterize the wtFN10 complex, where the angle ranges between 21° and 84° (S2 Fig in supporting information).

Comparable results were obtained with DynDom. [32,33] In two out of the three hFN10 replicas, DynDom fails to identify clusters of rotation vectors, therefore bound hFN10 is not captured by the algorithm as an independently moving domain. In the complex with wtFN10, comparing starting and final conformations, the principal rotation axis discriminates motions of the fibronectin with respect to the β3 subunit, with a rotation angle of around 55°.

**Fig 3:**
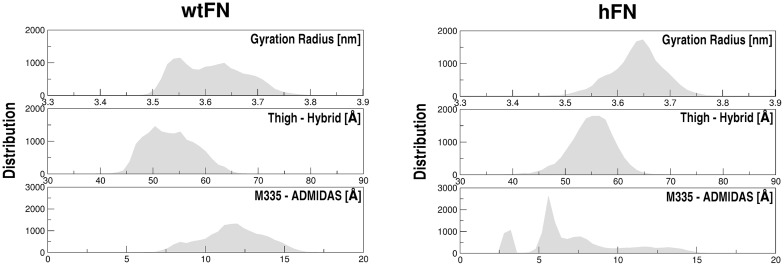
Gyration radius, end-end and Met335_β3_ (carbonyl oxygen) and Mn^2+^-ADMIDAS distances of wtFN10 and hFN10. Distributions refer to the full-length simulation time (3*0.5 μs = 1,5 μs per system). Wider populations indicate higher variability. See main text for the definition of Thigh-Hybrid (end-end) distance. See also S3 Fig).

The Radius of Gyration (Rg) and the evolution of the end-to-end distance of the thigh and hybrid domains (defined between the Cα of two terminal residues -namely I592_αv_ and R404_β3_ of thigh and hybrid domains, respectively) were next monitored as descriptors of integrin dynamic response to different ligands (Fig 3). Moreover, given the critical role of ADMIDAS in βA domain allostery [21] we also monitored the relative positions of the carbonyl oxygen of M335_β3_ and ADMIDAS Mn^2+^, since it was previously shown as a viable indicator of the closed-to-open transition in the eight steps atomic description of the RGD-induced opening occurring in the β3 subunit of αIIbβ3. [10] In the last opening steps (from state 6 to state 8) ADMIDAS ion shifts dragging along α1 helix as a rigid body with its coordinated D126_β3_ and D127_β3_ while M335_β3_ at the β6-α7 loop moves apart. [10]

Fig 3 compares the distribution of Rg, end-end distances and M335_β3_ –ADMIDAS distances for αvβ3-wtFN10 and αvβ3-hFN10 complexes for all replicas: a more dynamic behavior is consistently observed for wtFN10, indicated by the wider distributions of the distances between the thigh and hybrid domains, the Rg and the M335_β3_-ADMIDAS distances. In wtFN10 X-ray structure M335_β3_-ADMIDAS distance is 14.6 Å while in hFN10 is 2.9 Å. For the wtFN10 system the distribution shows a broad range of values around ~ 12.4 Å, while for hFN10 the distribution is more sharply centered around two values, 2.6 Å and 5.2 Å. Time-dependent evolution of M335_β3_-Mn^2+^-ADMIDAS distances together with representative integrin conformations are given in S3 Fig to account for the tails in the distribution analysis of the hFN10 system. Notably, even though some variations in the M335_β3_-Mn^2+^-ADMIDAS for the hFN system can transiently become similar to the wtFN system, these variations are not paralleled by the global rearrangement of the integrin.

Taken together, these first observations support the onset of different dynamic regimes in the two complexes, suggesting that the ability of the wtFN10 complex to explore a larger conformational ensemble may be linked to a more favorable tendency towards activation.

### Ligand-dependent integrin internal dynamics

The type of bound fibronectin and the consequent differences in the corresponding X-ray structures appear to have a profound influence on the dynamic evolution of the complexes. To gain fine-grained insights into the determinants of the dynamic differences we calculated the fluctuations of pairwise amino acid distances (Distance Fluctuation, DF) [34] in the MD trajectories of the two complexes (see Methods). Such measure has been previously shown to shed light on the effects of ligands on internal long-range pair coordination. [35] While intra-domain coordination is somewhat expected given the spatial proximity between amino acids, the analysis of coordination patterns between residues that belong to different domains can aptly highlight internal dynamic modulations that depend on the identity of the bound ligand.

**Fig 4:**
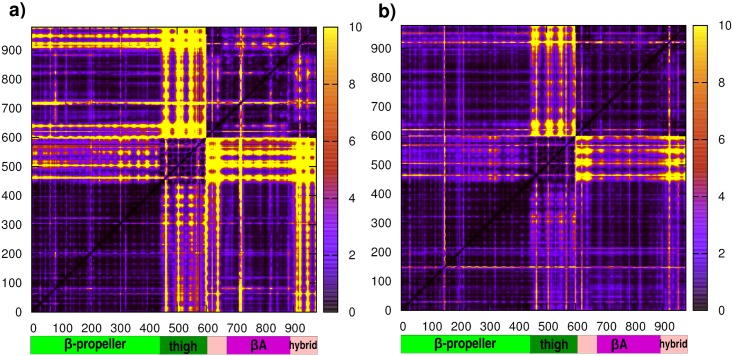
DF matrices. Distance fluctuations are averaged along full-length simulation time for a) wtFN10 and b) hFN10. Darker spots indicate low inter-residual fluctuation, while lighter stripes evidence highly flexible inter-distances. For clarity only αvβ3 domains are plotted. On x- and y- axes integrin residues are reported (diagonal matrix) and divided into αv (green) and β3 (pink) subunits.

The DF matrix for the complex with wtFN10 (Fig 4a) indicates more fluctuating internal dynamics. Regions of strong dynamic coordination (low fluctuation) alternate with regions where inter-residues distances show large fluctuation, i.e. low coordination. In particular, αv-thigh and β3-hybrid domains appear as the regions of greatest variation. In an interesting contrast, the presence of high-affinity FN (hFN10) reverberates in the increase of overall rigidity of the αvβ3 integrin, characterized by patterns of highly coordinated (low fluctuation) residue pairs that diffuse throughout the structure of the whole protein: in particular, in Fig 4b DF matrices show that thigh and hybrid domains of the αv and β3 are highly coordinated. Increased rigidity and coordination among different domains may oppose the onset of conformational changes required for integrin activation.

Additional information on the mechanistic determinants of different functional properties is obtained by analyzing the residue-based root mean square fluctuations of αvβ3 in the two complexes. Interestingly, this simple measure shows that the largest divergences occur at the αvβ3 interacting surfaces. In particular, fluctuations in the β-propeller-βA interfaces in the wtFN10 complex are much larger than in the case of the high-affinity complex. Averaged fluctuations and relative standard deviations for the three replicas per system are displayed in Supporting Information in S4 Fig.

Overall, these data indicate that the two different FN10 sequences trigger specific differential dynamic responses in the two complexes, evident at the level of macroscopic structural changes as well as at the level of microscopic internal coordination. Binding of wtFN10 or hFN10 at the interface between the integrin alpha and beta subunits may thus be linked to the onset of different functionally oriented conformational events.

It is necessary to underline here that MD simulations carried out on the uncomplexed αvβ3 as control show that the apo dynamics is less rigid than the hFN10 and more reminiscent of the dynamical character of agonist bound wtFN10 (S5 Fig). It is to be noted here that the apo system lacks LIMBS and ADMIDAS ions due to crystallization conditions.

### Interactions at the interface FN-αvβ3: hFN vs wtFN

The knowledge obtained so far on differential aspects of the αvβ3-fibronectin dynamics is complemented here by a comparative analysis of the fine specific interactions in the interface region that can be aptly used to make manifest the relatedness between the binding of different sequences flanking the RGD motif and the origin of specific functional dynamics.

**Fig 5:**
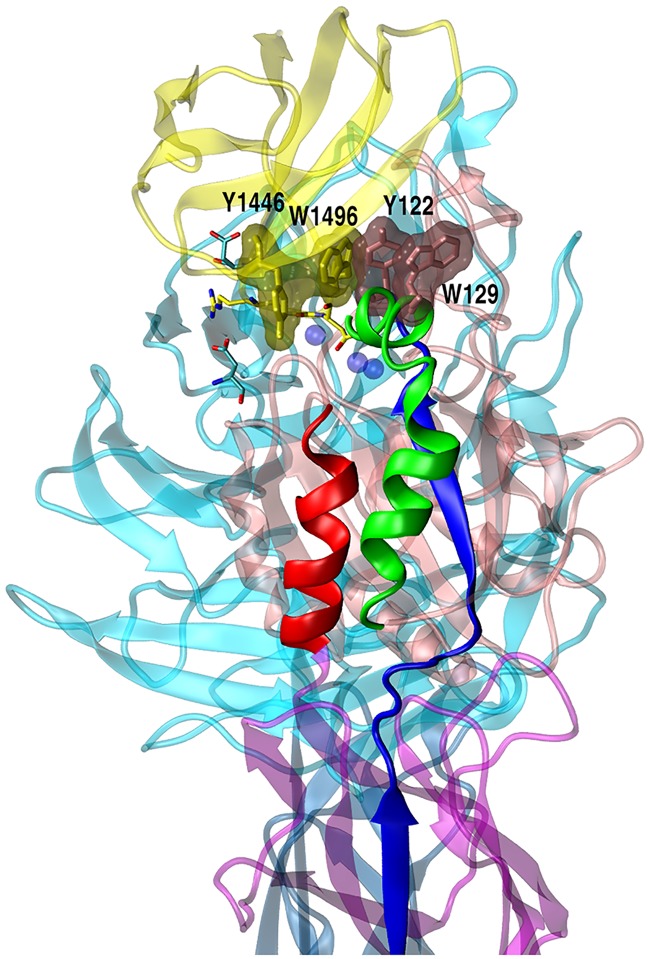
Close-up view of hFN10 complex. Important structural elements are indicated: α1 (green), α7 (red), strand β5 (blue). Mn^2+^ are displayed as transparent blue spheres. Ghost cartoons are used for αvβ3 (cyan and pink codes for αv and β3, respectively) and fibronectin (yellow). Ghost surface evidences W129_β3_, Y122_β3_, W1496_FN_ and Y1446_FN_ (from the right to the left). RGD-guanidinium and aspartic acids of the electrostatic clamp (namely D150_αv_ and D218_αv_) are also indicated as sticks. Note that the solvent-exposed Y122_β3_ belongs to β5 strand (blue) that spans the whole β3 subunit (see main text).

The high affinity -P**RGD**WNEG- sequence in hFN10 favors a characteristic placing of the RGD motif at the αvβ3 interface, in which fibronectin W1496_hFN_ is observed to limit the mobility of such fragment and of the whole hFN10-complex as a consequence: at the interacting surfaces, hFN10 shows extensive packing with bulky aromatic amino acids from the βA domain. At the beginning of the simulation, aromatic side-chains of Y122_β3_, W129_β3_, Y1446_hFN_ and W1496_hFN_ align to get closer to one another and form a tightly packed aromatic cluster. The stability of such association is validated by pairwise centroid distances and interplanar angle (θ) evolution along the full-length simulation time (1.5 μs) (Fig 5 and S6 Fig).

At the same time, this conformation permits the electrostatic clamp of the Arginine guanidinium of the RGD motif with D218_αv_, D150_αv_ and it favors the stabilization of the initial coordination of Mn^2+^ ions at LIMBS, ADMIDAS and MIDAS sites. Overall, the hydrophobic packing may 'direct' the onset of novel interactions, acting as the driving force for the stabilization in solution of the structure obtained by crystallography.

The combination of bulky and packed organization of residues, electrostatic stabilizing interactions and metal coordination sum up to impede movements in the immediate vicinity, limiting the motional freedom of hFN10 (Fig 2 and S1 Fig). The final consequence is that no conformational rearrangement is observed, and the original/crystallographic orthogonal orientation at the interface is conserved. Overall, in the hFN10 complex the conformation of the β3 subunit and the coordination at MIDAS/ADMIDAS are qualitatively similar to the S1 state of αIIβ3 integrin described by Springer [10] as a closed/inactive state, as already mentioned. It should be noted that this similarity is mostly qualitative, as the two integrins represent different systems, with different organizations and sequences.

In contrast, in wtFN10, the fibronectin domain bends upon βA domain providing access to dynamic states that lead to viable conformations alternative to the ones observed in the crystal structures. The Oδ1/Oδ2 Asp (RGD) coordination of the metal ion at MIDAS appears to be less stable than the correspondent one in hFN10 (S7 Fig). Furthermore, for wtFN10 the classical interaction between R1493_wtFN_ and D150_αv_ and D218_αv_ is broken to form a novel interaction with D219_αv_ while for hFN10 is mainly conserved. On the other hand, the coordination of D1495_FN_ of RGD motif to the MIDAS cation is kept in all replicas of both wtFN10 and hFN10 systems (S7 Fig).

Finally, in wtFN10 the sequence flanking the RGD motif, -G**RGD**SPAS-, cannot establish the hydrophobic association determined by the tryptophan indole (-P**RGD**WNEG-) in hFN10. [1,2] In wtFN10 Y1446_wtFN_ is engaged in interactions with αv chain (see S2 Table) while integrin W129_β3_(βA) is free to flip outward, populating an alternative rotameric state.

The higher conformational variability of W129_β3_(βA) in the complex with wtFN10 compared to hFN10 is clearly highlighted by structural cluster analysis (see Methods and S8 Fig). In the wtFN10 case, two major conformational clusters are visited, corresponding to the starting X-ray-like and flipped orientations of the side chain. The two conformations are reminiscent of the S7/S8 states defined by Springer and coworkers [10], respectively. In contrast, in hFN10 the indole side-chain is mostly exposed to the solvent in an arrangement overall similar to the starting conformation.

The observed α1-W129_β3_ rotamer switch in wtFN10 causes a general rearrangement in the interaction network by invading the space of the β6-α7 loop, consistent with the mechanism proposed by Springer and collaborators for the last steps toward αIIbβ3 opening path. [10] In hFN10, W129_β3_ preferentially stabilizes around the starting conformation, thanks to extended packing with hFN10 W1496_hFN_ (to be noted that this residue corresponds to S1496 in wtFN10). Overall, in the wtFN10 complex the conformation of the β3 subunit and the coordination of MIDAS/ADMIDAS are qualitatively similar to the S7/S8 states of αIIβ3 integrin (3ze1.pdb, chain B) described by Springer and coworkers as an open/active state. [10]

These structural observations indicate that the behavior of the wtFN10-integrin complex is much richer than that of hFN10. It must be noted here that we are not describing the opening transition of integrin, but we are observing the possibility for the complex with wtFN10 to explore a diverse range of local interaction networks and conformational states of specific residues that are compatible with different activation states described by Springer and coworkers [10], albeit for a different yet related system. Such considerations are consistent with the initial hypothesis that integrin may react to binding a protein ligand primarily via a fine-tuned reorganization of intra-protein interaction networks and dynamic states, which can eventually be connected to the onset of large scale motions.

Next, we calculated hydrogen bonds and salt bridges at the interacting surfaces of FN10 and integrin. Apart from aspartic acids from the β-propeller (D150_αv_, D218_αv_) that coordinate the arginine guanidinium of the RGD motif (R1493_FN_), novel interactions are established during the simulations that can be used to discriminate the two complexes. Compared to the X-ray starting structure, only during the wtFN10 complex simulations we observed the formation of new salt bridges with the β-propeller residues. Important electrostatic interactions are R1448_wtFN_-D218_αv_, R1445_wtFN_-D218_αv_, -D150_αv_ and -D148_αv_ and R248_αv_-E1462_wtFN_. Two different salt bridges are formed with β subunit of both systems: R1448_wtFN_-E312_β3_ and R1445_hFN_-D251_β3_ (S2 Table).

In wtFN10 simulation, the crystallographic Y122_β3_ backbone bond with the Oδ2 oxygen of Asp (RGD) is transient and it is lost in the first frames of conformational rearrangement. Thus an outward movement of the tyrosine exposes it to the solvent. In contrast, in hFN10 the indole moiety of the W flanking residue of the RGD-optimized sequence -P**RGD**WNEG- freezes Y122_β3_ at the top of α1 helix via hydrophobic packing as already illustrated (Fig 5).

Notably, the hydrogen bond between Asp (RGD) and Y122_β3_ (and consequently the β1-α1 loop) seems to play a critical role in regulating the lifetime of the principal RGD-αvβ3 bond (Asp-MIDAS) by shielding it from free water molecules, as already reported by Vogel and collaborators. [21] Consistently, previous docking studies have conferred a critical role to Y122_β3_ in the ligand-receptor recognition process. [36,37]

Another important interaction between hFN10 and β3 concerns D251_β3_. This amino acid stably forms a salt-bridge with R1445_hFN_. During the simulation of the wtFN10 system D251_β3_ does not form any H-bonds or salt bridges with the receptor and the same arginine can be engaged by different amino acids from the αv domain in the wtFN10, e.g. D218_αv_-R1445_wtFN_ (see S2 Table and S9 Fig). Due to its nature and position, such interaction (alternating between α and β chain) is crucial to stabilize the orientation of the fibronectin onto the integrin ectodomain.

Not surprisingly, the N215_β3_-D1495_FN_ (RGD) hydrogen bond (one of the crystallographic interactions) is the only conserved contact displayed by all replicas, and in hFN10 it is also stably engaged by the other carboxylate oxygen of the aspartic acid of the RGD. Such interaction is observed in approximately 23% of the total simulation time (1,5 μs) in the wtFN10 whereas it is present in the 38% for the hFN10.

This observation can be related to the swing-out mechanism of the hybrid domain. The RGD coordination of this asparagine, located in the loop connecting helices α2 and α3, is incompatible with the α1-β1 motion and then with the hinge opening. Recently, similar conclusions were reported for not-inducing swing-out of small isoDGR containing peptides. [24]

Next, we analyzed the structural responses of the MIDAS and ADMIDAS binding sites to the presence of either FN10 form. Experimentally, the difference between open and closed conformation at the βA-hybrid domain interface is translated into a ~3 Å displacement of the MIDAS and ADMIDAS-coordinating β1-α1 loop of the βA domain, which alters affinity for RGD-partner binding by ~1,000-fold in αIIbβ3. [10] ADMIDAS metal ion moves toward the MIDAS metal ion also in α5β1. [38] These rearrangements are parallel to βA domain opening to a high-affinity state.

To this end, we monitored Mn^2+^ distance at MIDAS and ADMIDAS and correlated it with the β-hybrid opening. In good agreement with experimental observations, we observe smaller MIDAS-ADMIDAS Mn^2+^ distances for wtFN10 simulations. In particular, shorter Mn^2+^- Mn^2+^ spacing coincides with the largest Thigh-Hybrid relative distortion, at long-range distances (S10a Fig). This is not the case of hFN10 where MIDAS-ADMIDAS separation is conserved and settled around a longer distance than in the *wild type* (S10b Fig). From the cumulative distribution of Mn^2+^-Mn^2+^, it is evident that hFN10 is characterized by a dominant peak centered at 8.5 Å, with a queue of less populated bins at higher distances. In the case of wtFN10, the distribution is wider and with bins of comparable populations spanning a larger set of distances, reaching smaller values (~6 Å). As we have already commented above, observed local rearrangements do not necessarily associate to global conformational changes (S3 Fig).

Overall, analysis of binding networks shows once more that the wtFN10 explores diverse sets of interactions with αvβ3, favoring the population of different conformational ensembles than the ones determined by hFN10. Globally, wtFN10 may determine a deviation from the original crystal structure leading to the integrin opening motion linked to activation, while hFN10 stabilizes the complex in the closed conformation.

## Discussion

### Dynamical and functional Implications of integrin-fibronectin interactions

hFN10 acts as a pure antagonist of αvβ3 and lacks the partial agonism that is often observed in other protein ligands that exploit RGD as a recognition motif, as well as RGD-based peptidomimetics. [3]

At the β-propeller-βA interface FN inserts into the binding cleft by orienting its RGD motif to enable Asp(Oδ1/Oδ2) to coordinate the metal ion at MIDAS site. The binding of the recognition triad occurs across the αv and β3, where the guanidinium of the Arginine (RGD) shifts between D218_αv_ and D150_αv_ of the β-propeller domain of the αv subunit.

While RGD binding takes place in a similar fashion in the two complexes, dissimilar orientations of the full-length fibronectins, wtFN10 and hFN10, on the top of the integrin determine different local interaction networks that reverberate in dissimilar structural rearrangements (Fig 2 and S2 Fig): wtFN10 rotates upon the βA domain in the first nanoseconds of the simulations, making several stable novel contacts with superficial amino acids from both integrin chains. In contrast, hFN10 persists in its original orientation for the full trajectory, establishing interactions mainly with the βA lobe. Differential effects are arguably attributable to changes in the sequences flanking the RGD recognition motif.

X-ray resolution of hFN10-αvβ3 complex shows a π-π edge-to-face interaction for Y122_β3_ and W1496_hFN_. Along the simulation, starting on the top of helix α1, we observe an increased association of highly bulky and aromatic amino acids, that form a stable and tightly packed hydrophobic core at the interacting surfaces, involving W129_β3_ and Y122_β3_ from βA and Y1446_hFN_ and W1496_hFN_ from high affinity fibronectin (Fig 5 and S6 Fig).

The crystallographic distance between Y122_β3_ and W1496_hFN_ centroids is 4.9 Å while the interplanar angle θ is 25.8°.

The relative orientation and distance between these residues is well preserved along the trajectory. Distances between their centroids and the distributions of angles between their correspondent planes (θ) can be considered in the range of general hydrophobic π-stacking for aromatic residues (distance between 4.9 and 10.4 Å, θ angle between 1.2° and 89.9°, for stacked and T-shaped arrangements). [39–42]

In the wtFN10 complex, Y122_β3_ forms a π-cation interaction with R214_β3_ of the βA chain and this packing is stably preserved along the simulation (distance between 4.6 and 6.9 Å). Moreover, corresponding integrin-W129_β3_ in the wtFN10 complex results very flexible, pointing alternatively towards the solvent or towards the interior of the protein (see S8 Fig). Large changes in position and rotamer of this residue have been extensively discussed and linked to the activation opening described for αIIbβ3. [10]

These data point to the role of the high-affinity RGD sequence in hFN10 in favoring the characteristic orthogonal docking of fibronectin on the top of αvβ3. The stable orientation and interactions of the hFN10 domain rigidify the whole complex, increasing internal coordination throughout the α and β subunits of the integrin. In this framework, the lack of the bulky tryptophan chain flanking the RGD region in wtFN10 can be considered as the determinant for the conformational re-organization observed in the wtFN10-αvβ3 complex. In our model, such a voluminous amino acid at the surface of hFN10 may prevent large movements and therefore represents the lock of the “activation” reaction, freezing and screening Y122_β3_ from solvation, in line with previous research. Structural and mutational studies support a critical role for the novel W1496_hFN_-Y122_β3_ π-π interaction in 'freezing' the integrin in an inactive conformation. [3]

The different interaction networks and microscopic conformational dynamics may represent the molecular determinants for the observed functional differences between the two complexes.

Together, these data point to αvβ3 functioning in specific ways determined by the sequences and recognition motifs of endogenous protein ligands. A key question in elucidating integrin activation relates to what determines the onset of specific functionally oriented motions and the ability to predict their consequences: this would allow the distinction of the role of the ligand as an agonist or an antagonist.

In our study, we distinguish and characterize opening motions vs. conformational stabilization as a function of differences in the ligand binding sequences: in this frame of thought, the extensive and stable packing determined by the tryptophan side chain in hFN10 acts as a blocker for the initial series of conformational events necessary for integrin activation, namely β3 opening and overall reorganization of domain distances and orientations. In contrast, the absence of tryptophan in wtFN10 determines a different set of contacts that translates into a much more pronounced internal flexibility of the integrin domains paralleled by the ability to explore a wider portion of conformational space, which can ultimately favor integrin activation. Our results also highlight the role of the interaction networks of Y122_β3_ as the driver controlling conformational changes: this tyrosine is located at the top of the β5 strand that runs across hybrid and βA domains of the β3 subunit and the direct link to RGD-binding (Fig 5). Indeed, while the RGD binding site (namely, aspartic acids from the β-propeller—devoted to the electrostatic clamp—and metal ions within the βA-coordinated by Asp (RGD) carboxyl oxygen) is almost identical in the low-affinity and high-affinity form of the integrin, large conformational changes are seen at the hybrid lobe upon activation and, more precisely, at the hinge angle between βA and hybrid domains. [21] In this context, the “hinge opening” is considered as the first step that initiates the global genu-extension of the cytoplasmic tails. In fact, disclosing of the β-tail can only occur after the extension of the hybrid and βA domains, induced by the hinge opening.

On these bases, we can consider the mechanism of ligand-controlled (de)activation of integrin in the light of allosteric control concepts. The integrin binding pocket is conserved and serves to anchor the incoming ligand. The pocket interactions with different ligand sequences determine the accurate re-positioning of the FN domains: specific residues flanking the common RGD recognition motif in the two FNs modulate interactions with the integrin, supplying the critical foundation that allows the Y122_β3_. The residues of either FN10 interacting with Y122_β3_ act then as the driver residues that control the shifts of integrin population from the inactive (hFN10-bound) to the (partially) activated state (wtFN10-bound) through a specific reorganization of pre-existing interaction networks around Y122_β3_. We thus speculate that the extent of reorganization around the integrin binding site may determine the shift towards inactive or active states. The mechanism of stabilization of functional states differs as a function of the sequences of the ligands, and antagonism is determined by the presence of bulky, aromatic moieties flanking RGD that optimally pack in the integrin recognition site and consequently block hinge opening and domain reorganization. Agonism is favored by the absence of such flanking motifs, which allows more conformational freedom and pushes integrin towards the active conformation.

Delivering related but independent set of information, PCA and DF analyses can be combined to capture the main determinants of the differential conformational dynamics of the integrin induced by different ligand: the former characterizing large-scale collective motions, the latter identifying the sub-blocks and motifs that sustain the 3D fold organization necessary for activity. In addition, to further support the hypothesis that different interaction patterns at the binding site can trigger a differential global rearrangement of the integrin complex, we applied the linear mutual information method to unveil concerted correlations. This method can detect correlated motion regardless of the relative orientation and includes nonlinear contributions. [44] This analysis (S11 Fig) corroborates previous observations indicating that wtFN10 and hFN10 binding determine different dynamic responses of the integrin. In this framework, cross-correlations are markedly increased when hFN10 is bound at the αvβ3 interface. Moreover, correlations diffuse throughout the 3D structure. In the case of wtFN10, mutual information-based analysis shows that the mechanism for transmission stops at the local level, whereby high coordination appears to entail primarily residues that are proximal to the ligand. In the remainder of the integrin, higher coordination patterns appear to involve intra-domain relationships among residues. In contrast, in the case of hFN10, high mutual information relationships between residues span the whole complex “overcoming” the subdomains structural organization. Changes in correlations again are in agreement with previously discussed dynamical features observed during MD simulations.

Furthermore, it is important to emphasize here that quantitatively sampling the conformational changes at the basis of integrin activation/blockage by unbiased atomistic simulations is extremely demanding. Besides the use of pulling or targeted MD simulations which gave unprecedented insight into the basis of conformational changes [21–23], we hypothesize that such conformational changes cannot be simply described by one single reaction coordinate or by a combination of a limited number of simple reaction coordinates for metadynamics-type simulations. Indeed, we think that domain rotations and reorganizations should be taken into account.

In conclusion, the type of fine atomistic interactions and their action on microscopic conformational changes can help provide a molecular explanation of observed inhibiting vs. activation effects. Interestingly, we have shown that the comparative analysis of a combination of global measures (overall flexibility, large scale domain rearrangements) and of local structural environment variations in the recognition regions can reflect the difference between induced active and inactive states. Characterizing the determinants of microscopic dynamics from MD simulations can help rationalize the onset of functional protein motions, that can be linked to experimentally-observed structural and functional modifications.

In conclusion, these considerations may be useful in the design of small molecule modulators of the function of αvβ3 integrin function: we propose that it may be possible to realize peptidomimetics containing the RGD sequence in an optimal arrangement for stable binding, whose role as antagonist or agonist can be modulated by the type and stereoelectronic properties of the RGD-flanking groups.

By comparing the results of MD simulations with the determinants of integrin modulation induced by different FN sequences, we can investigate the effect of the aromatic substituent at the scaffold nitrogen atoms (exploring bulky side chains of aromatic amino acids) and of the recognition sequence (examining RGD-like motifs) on αvβ3 integrin internal dynamics. [44–45] The identification of peptidomimetic ligands able to efficiently mimic the behavior of the high affinity fibronectin mutant (underlying pure antagonism) could lead to the generation of new compounds that are unable to promote integrin activation and thus can act as pure antagonists.

In particular, the presence of large aromatic moieties may aptly block integrin opening, providing a new generation of real antagonists. [46–50] This hypothesis is supported by the recent observation that the modification of the barbourin KGDW sequence (venom disintegrin) led to the development of a cyclic hexapeptide (eptifibatide) that demonstrated high affinity for αIIbβ3. [46]

## Methods

### MD simulations

Molecular dynamics simulations were carried out on αvβ3-wtFN10 and αvβ3-hFN10, and on the unbound form of the integrin. Starting models were retrieved from Protein Data Bank with access number 4MMX, 4MMZ, and 1JV2, respectively.

Integrin extracellular domain was cut at the Thigh domain of the αv, including β-propeller and Thigh domains (residues 1–599) and at the β-hybrid domain of the β3, including βA and hybrid domains (residues 55–434); whereas fibronectin type-III domain 10 comprises residues 1417–1507.

To the best of our knowledge, no previous simulations have been carried out on the β-propeller—Thigh full-length domain of the αv chain. [21,51]

Wild-type fibronectin (wtFN10) tripeptide RGD-enclosed sequence -GRGDSPAS- is replaced by -PRGDWNEG- in high affinity fibronectin (hFN10) in X-ray structures.

Because Mn^2+^ ions are known to regulate integrin activation and are present under physiological conditions, crystallographic Mn^2+^ are kept at the three integrin metal ion binding sites as well as at the β-propeller domain.

The MD simulation package Amber v12 was used to perform computer simulation applying Amber-ff99SB*ildn force field. [52] The two systems were solvated, in a simulation box of explicit water molecules (TIP3P model), [53] counter ions were added to neutralize the system and periodic boundary conditions imposed in the three dimensions. Mn^2+^ ions were modeled based on hydration free energy parametrization derived from Musco et al. [24] Final simulated systems are made of ~ 200 000 atoms.

After minimizations, systems were subjected to an equilibration phase where water molecules and protein heavy atoms were position restrained, then unrestrained systems were simulated for a total of 3 microseconds, in a NPT ensemble; Langevin equilibration scheme and Berendsen thermostat were used to keep constant temperature (300 K) and pressure (1 atm), respectively. Electrostatic forces were evaluated by Particle Mesh Ewald method [54] and Lennard-Jones forces by a cutoff of 8 Å. All bonds involving hydrogen atoms were constrained using the SHAKE algorithm. [55]

To enhance sampling three independent replicas of 500 ns (3*0.5 μs = 1.5 μs) were run for each system with different initial velocities.

Figures are rendered using VMD. [56]

### Distance fluctuation analysis

We computed distance fluctuations, DF_ij_, along simulations to assess the intrinsic flexibility of proteins. Given r_ij_ the distance between Cα atoms of residues i and j:

$${\text{DF}}_{\text{ij}}=\;<{\left({\text{r}}_{\text{ij}}-<{\text{r}}_{\text{ij}}>\right)}^{2}>$$

distance fluctuation, DF_ij_, is defined as the time-dependent mean square fluctuation of the distance r_ij_, where the brackets indicate the time-average over the trajectory. DF is calculated for any pair of Cα along simulation time. Low DF values indicate highly coordinated residues. [34]

### Collective motions analysis

Based on the assumption that the major collective modes of fluctuation could be linked to protein function (Essential Dynamics [30]), MD simulations have been analyzed by means of principal components analysis (PCA). This method recovers the modes that produce the greatest contributions to the atomic root mean square deviations in the given dynamic ensemble. Thus large-scale collective motions can be collected, as well as the extreme conformations of the system along the simulation, providing information of time-dependent transitions. Additionally, we carried out linear mutual information as introduced by the Grubmuller group. [43,57] See supporting material for details.

### Domain axes and torsion angles

Definitions of rotation axes and torsion angles around these axes help to quantify conformational changes. Principal axes were determined for wtFN10 and hFN10 β3 integrin domains throughout the simulation time to follow structural transitions. Measurements were obtained by UCSF Chimera package. [31]

### DynDom description

Protein domains can be determined from the difference in the parameters governing their quasi-rigid body motion, and in particular their rotation vectors. Given a structure, by superimposing each main-chain segment in its initial conformation onto its final conformation by least-squares best fitting, we can define the displacement vectors representing the rigid body displacement of the segment. A K-means clustering algorithm is used to identify dynamic domains from clusters of rotation vectors corresponding to main-chain segments. The program DynDom takes two conformations, the initial and the final state, and determines dynamic domains on the basis of the conformational change. [32–33]

### Rotamers cluster analysis

Cluster analysis was performed over the region of α1(βA) including W129 (aa. M124-I131) of the β3 chain, using Gromos method for clustering, described by Daura et al. [58]: To find clusters of structures in a trajectory, the RMSD of atom positions between all pairs of structures is determined. For each structure the number of other structures for which the RMSD is 0.2 nm or less is calculated. The structure with the highest number of neighbors is taken as the center of a cluster, and forms together with all its neighbors a (first) cluster. The structures of this cluster were thereafter eliminated from the pool of structures. The process was repeated until the pool of structures was empty. In this way, a series of non-overlapping clusters of structures was obtained. Central structure of each cluster is provided. 5000 time frames per replica (15000 per system) are used in the calculation.

## Supporting Information

S1 Text Essential dynamics and linear mutual information. (DOCX)

S1 Table PCA sampling. The inner product of the essential eigenvectors of the first half with the essential eigenvectors of the second half of each simulation and cosine content for replica is shown.
(DOCX)

S2 Table List of hydrogen bonds and salt bridges between fibronectin and αvβ3 in the wild-type (wtFN10) and high-affinity (hFN10) complexes. Bonds are calculated considering full-length FN against αvβ3. Amino acids of the αv chain (β-propeller) are indicated in bold. * indicates contacts present in the x-ray structure. % is the fraction of time (1,5 μs per system) where the interaction is present. Only interactions with % higher or equal to 1 are reported. RGD motif numbering is R1493,G1494,D1495.
(DOCX)

S1 Fig ED analysis. a) Time-dependent projections of wtFN and hFN onto the first two principal modes per replica. b) Projection of wtFN and hFN onto the first two principal modes of the three simulations. Calculations are carried out on C-alpha atoms (979 particles) of αvβ3. Reference structure used for the fitting of either systems is integrin in the wtFN complex (pdb code: 4MMX). The two eigenvectors account for the 55% (wtFN10) and 46% (hFN10) of the total variance of the simulations.
(TIF)

S2 Fig Rotation axes of a) wtFN10 and b) hFN10 complexes. β3 (pink) and Fibronectin (yellow) domains are shown as cartoons in the starting conformation (t = 0) and corresponding principal axes are displayed. Blue arrows indicate the fibronectin torsion angle along the simulation time (replica #1). For clarity, only β3 subunit is shown and the orientation of the two domains in a) and b) displays the maximal value of the distortion. Torsion angles ranges between 21° and 84°(a) and between 56° and 82° (b). c) Time-dependent evolution of the rotational angle described by the principal axes of β3 and FN domains for wtFN10 and hFN10 in the three replicas. See main text.
(TIF)

S3 Fig Met335_β3_-Mn^2+^ (ADMIDAS) distance. Time evolution series are plotted for each replica (0.5 μs) of wtFN (a) and hFN (b) systems. Integrin conformations corresponding to representative distance values are displayed. For hFN we report structures relative to two extreme distance values.
(TIF)

S4 Fig Root mean square fluctuation. Root mean square fluctuations are plotted per residue (r) and averaged for the 3 replicas along simulation time for wtFN10 (left) and hFN10 (right) systems. Major differences are visible at β3 chain (starting at r = 600). For clarity, fibronectin domain has been omitted. Error bars represent standard deviation.
(TIF)

S5 Fig Apo αvβ3 dynamics (1JV2). a) ED analysis. Projection of the uncomplexed αvβ3 onto the first two principal modes of the simulation. Calculations are made on the C-alpha atoms. b) DF matrix. Distance Fluctuations are averaged along 500 ns simulation time. Darker spots indicate low inter-residual fluctuation, while lighter striper evidence highly flexible inter-distances.
(TIF)

S6 Fig Hydrophobic cluster. a) Pairwise centroid distances and b) interplanar angle (θ) evolution between Y1446_hFN_, W1496_hFN_, Y122_β3_, W129_β3_ of the hydrophobic cluster along simulation time (0.5 μs * 3 replicas of hFN10). Red diamonds indicate Y122 _β3_-W1496_hFN_ packing evolution confirming π- stacking described in ref 3 and discussed along the main text. Note that amino acids are considered paired for centroid distances < 12 Å, while interplanar angle distribution (between 0° and 90°) accounts for stacked and T-shaped conformations.
(TIF)

S7 Fig Fluctuation of Mn2+ at MIDAS. Coordination of Mn2+ at Midas site by carboxylic oxygens- Oδ1 (black) and Oδ2 (red)—from aspartic acid of RGD for individual replica. High flexibility is shown by wtFN10 coordination shell (left panel) compared to the more stable hFN10 system.
(TIFF)

S8 Fig Cluster analysis of wtFN10 and hFN10 MD trajectories. Close-up view of the βA (β3) that directly contacts fibronectin (transparent cartoons). α1 is shown as pink solid cartoons and α1-W129_β3_ rotamers in sticks. a) Central structure of each cluster for wtFN10 (replica #1) is displayed starting from red (starting frames of the simulation and 0.56% of representativeness) to the gray/blue rotamers (final frames and accounting for the remaining 99.4% of the population). b) Central structure of the unique cluster found for hFN10. Cluster analysis was performed over the region of α1(βA) enclosing W129 (aa. M124-I131) of the β3 chain, using an RMSD cutoff of 0.3 and selecting Gromos method for clustering. [4] 5000 time frames per replica were analyzed.
(TIFF)

S9 Fig Example of fibronectin-integrin interactions. a) Hydrogen bond evolution: wtFN10 D218_αv_-R1445_FN_ in the top panel, and hFN10 D251_β3_-R1445_FN_ in the bottom panel. b) Spatial rearrangement of fibronectin in the complex. αvβ3 is in cyan and pink cartoons while FN10 domain is colored yellow. Interacting amino acids are shown in sticks using the same color code of the correspondent subunit.
(TIFF)

S10 Fig Midas-Admidas displacement. a) Relationship between Mn^2+^-Mn^2+^ distances at Midas and Admidas and long-range conformational change. Solid ribbons for αvβ3 and blue spheres for Mn^2+^ and transparent ribbons and white spheres are used for the two extreme structures of the structural rearrangement. b) Cumulative distribution of Mn^2+^-Mn^2+^ interdistances at Midas and Admidas binding sites. Data sets refer to full-length simulation time (1,5 μs) for wtFN10 and hFN10 systems. The main difference between wild type and high affinity FN10 is to be found in the width and height of the bars in the histogram. In this respect it appears that hFN10 is characterized by a dominant peak centered at 8.5 Å, with a queue of less populated bins at higher distances. In the case of wtFN10, the distribution is wider and with bins of comparable populations spanning a larger set of distances.
(TIFF)

S11 Fig Linear mutual information analysis. Comparison of generalized correlation coefficient matrices for wtFN10 (a) and hFN10 (b). Data represent average matrices of the three replicas per system (calculated on c-alpha atomic coordinates. c) 3D mapping of the most correlated blocks of hFN10 (color scale of red boxes in b is proportional to correlation). Green box refers to FN domain. The generalized correlation can detect correlated motion regardless of the relative orientation and includes nonlinear contributions. A density estimator nearest-neighbor parameters k = 6 was applied.
(TIF)
