# Supplementary material for: High Affinity vs. Native Fibronectin in the Modulation of αvβ3 Integrin Conformational Dynamics: Insights from Computational Analyses and Implications for Molecular Design
Source: PLoS Comput Biol. 2017 Jan 23;13(1):e1005334. doi: 10.1371/journal.pcbi.1005334 (PMC5293283; doi:10.1371/journal.pcbi.1005334)
Supplement: S1 Text — (DOCX) [file pcbi.1005334.s001.docx]

**Supporting information**

**ED analysis.**

Two PCA analyses are carried out.

1) C-alpha atoms of the full-length complex (integrin+FN = 1070 particles) are used for the PCA, and the crystallographic X-ray structures of the *wild-type* complex, namely 4MMX, is used as reference for the wtFN10 and hFN10 trajectories. 5000 snapshots per trajectory per system (stride = 100 ps) are projected on the essential subspace described by the first 2 eigenvectors responsible for the maximum variation in conformation observed along molecular simulation. In this way, it is possible to trace the conformational space of the wtFN10-complex spanned by the hFN10-complex. The two eigenvectors account for the 56% (wtFN10) and 48% (hFN10) of the total variance of the simulations. Convergence of the sampling has been assessed using the inner product (overlap) of the essential subspaces between the first and the second half of the simulation. Overlap values close to zero indicate that sampled subspaces are substantially different and low cosine content indicate that largest fluctuations are not due to random diffusion.[1-2] In table S1 the inner product of the essential eigenvectors of the first half with the essential eigenvectors of the second half of each simulation and cosine content for replica is shown.

**Linear Mutual Information**

Correlated motions in proteins are calculated by the cross-correlation method developed by Lange et al.,[6] where correlations between residues motions overcome the problems of the commonly employed Pearson coefficient. This measure, in fact, is dependent on the relative orientation of the atomic fluctuations and, treating correlations in a quasi-harmonic (linear) approximation, is unable to detect nonlinear correlations, with marked artifacts in the quantification of motion correlations. In generalized correlation approach, the assumption rests on the fundamental definition of independence of random variables. The joint probability distribution *p*(**x**) of N random variables **x***_i_* with *i* = 1,…,*N*, equals the product of their marginal distribution *p_i_*(**x**_i_) if and only if the components **x***_i_* are independent, i.e. uncorrelated. Therefore, mutual Information (MI) gives the ensemble-averaged deviation from the uncorrelated distribution:

$$I\left[ \boldsymbol{x}_{\boldsymbol{1}},\boldsymbol{x}_{2},\ldots,\boldsymbol{x}_{\boldsymbol{N}} \right]=\int p\left( \boldsymbol{x} \right)ln\frac{p(\boldsymbol{x})}{\prod_{i=1}^{N} p_{i}(x_{i})}d\boldsymbol{x}$$

For a more familiar interpretation, a Pearson-like coefficient derived from MI, r_MI_, is defined:

$$r_{MI}\left[ \boldsymbol{x}_{i},\boldsymbol{x}_{j} \right] = \left\{ 1-exp\left( -2I\left[ \boldsymbol{x}_{i},\boldsymbol{x}_{j} \right]/d \right) \right\}^{-1/2}$$

The coefficient ***r***_MI_ is zero for fully uncorrelated variables and assumes values up to 1 for fully correlated variables.

Here, the information content is calculated using the atomic positions fluctuations derived from MD simulations by means of k-nearest neighbor distance algorithm, as implemented in GROMACS[(Hess, Kutzner, Van Der Spoel, & Lindahl, 2008)].

**References**

1. B. Hess. Similarities between principal components of protein dynamics and random diffusion. *Phys Rev E*. **2000**, *62*(6):8438–8448.
2. B. Hess. Convergence of sampling in protein simulations. *Phys Rev E.* **2002**, *65*(3):031910.
3. G. B. Mcgaughey, M. Gagné, A. K. Rappé. π -Stacking Interactions. *J Biol Chem*. **1998**, 273(25):15458-15463.
4. X. Daura, B. Jaun, D. Seebach, W.F. van Gunsteren, A.E. Mark. . Reversible peptide folding in solution by molecular dynamics simulation. *J Mol Biol.* ***1998****.* 280(5):925–32.
5. B. Hess, C. Kutzner, D. Van Der Spoel, E. Lindahl. GROMACS 4: Algorithms for highly efficient, load-balanced, and scalable molecular simulation. *J Chem Theory Comput,* **2008***.*4(3):435-47.
6. O. Lange, H. Grubmuller. Generalized correlation for biomolecular dynamics. *Proteins.* **2006**, 62(4):1053-61.
