# Supplementary material for: High Affinity vs. Native Fibronectin in the Modulation of αvβ3 Integrin Conformational Dynamics: Insights from Computational Analyses and Implications for Molecular Design
Source: PLoS Comput Biol. 2017 Jan 23;13(1):e1005334. doi: 10.1371/journal.pcbi.1005334 (PMC5293283; doi:10.1371/journal.pcbi.1005334)
Supplement: S1 Table — The inner product of the essential eigenvectors of the first half with the essential eigenvectors of the second half of each simulation and cosine content for replica is shown. (DOCX) [file pcbi.1005334.s002.docx]

**S1 Table.**

|  |  | Inner product  (0-250 ns *vs* 250-500 ns) | Cosine content |
| --- | --- | --- | --- |
| ***wtFN*** | Replica #1 | 0.38 | 0.76 |
|  | Replica #2 | 0.25 | 0.52 |
|  | Replica #3 | 0.39 | 0.15 |
|  |  | |  |
| ***hFN*** | Replica #1 | 0.43 | 0.23 |
|  | Replica #2 | 0.44 | 0.71 |
|  | Replica #3 | 0.47 | 0.45 |
