# Supplementary material for: High Affinity vs. Native Fibronectin in the Modulation of αvβ3 Integrin Conformational Dynamics: Insights from Computational Analyses and Implications for Molecular Design
Source: PLoS Comput Biol. 2017 Jan 23;13(1):e1005334. doi: 10.1371/journal.pcbi.1005334 (PMC5293283; doi:10.1371/journal.pcbi.1005334)
Supplement: S2 Table — Bonds are calculated considering full-length FN against αvβ3. Amino acids of the αv chain (β-propeller) are indicated in bold. * indicates contacts present in the x-ray structure. % is the fraction of time (1,5 μs per system) where the interaction is present. Only interactions with % higher or equal to 1 are reported. RGD motif numbering is R1493,G1494,D1495. (DOCX) [file pcbi.1005334.s003.docx]

**S2 Table**

| **Hbonds** | | | | | **Salt Bridges** | | | |
| --- | --- | --- | --- | --- | --- | --- | --- | --- |
|  | **wtFN** | **%** | **hFN** | **%** | **wtFN** | **%** | **hFN** | **%** |
| **1** | **R1445-D218** | 33.5 | **R1493-D218*** | 34.8 | **R1493-D218*** | 46.0 | **R1493-D150** | 42.6 |
| **2** | **R1493-D218*** | 31.2 | **R1493-D150** | 22.7 | **R1493-D150*** | 45.2 | **R1493-D218*** | 30.7 |
| **3** | **R1493-D150** | 23.9 | **R1493-E123** | 6.3 | **R1445-D218** | 31.3 | R1445-D251 | 30.2 |
| **4** | **R1445-D148** | 23.3 | **R1493-D148** | 4.5 | **R1445-D150** | 30.0 |  |  |
| **5** | **R1493-D219** | 16.3 | **T1497-Y178** | 2.6 | **R1445-D148** | 25.5 |  |  |
| **6** | **R1445-D150** | 13.5 | **S1417-D150** | 4.1 | **E1462-R248** | 21.0 |  |  |
| **7** | **Y1446-D150** | 11.4 | **T1443-I216** | 1.2 | **R1493-D219** | 15.8 |  |  |
| **8** | **R1448-D218** | 4.6 | **S1417-D148** | 1.0 | **R1448-D218** | 14.8 |  |  |
| **9** | **Y1446-D148** | 3.6 | D1495-N215 | 38.3 | R1448-E312 | 10.8 |  |  |
| **10** | **R1493-F177** | 2.0 | R1445-D251 | 19.8 |  |  |  |  |
| **11** | D1495-N215 | 23.0 | E1462-K125* | 13.3 |  |  |  |  |
| **12** | D1495-S123 | 18.4 | E1462-Y122 | 10.2 |  |  |  |  |
| **13** | V1460-N316 | 10.9 | T1497-R216 | 9.8 |  |  |  |  |
| **14** | S1499-D126 | 9.5 | Y1488-D179 | 7.8 |  |  |  |  |
| **15** | A1498-D126 | 8.9 | R1448-M180 | 7.1 |  |  |  |  |
| **16** | R1448-E312 | 7.8 | T1497-N215 | 4.3 |  |  |  |  |
| **17** | S1499-D127 | 7.5 | T1464-D336 | 3.9 |  |  |  |  |
| **18** | T1450-E312 | 7.1 | E1462-T182 | 2.6 |  |  |  |  |
| **19** | V1460-Q319 | 6.6 | R1445-N313 | 2.5 |  |  |  |  |
| **20** | T1443-P176 | 5.1 | W1496-N215 | 2.1 |  |  |  |  |
| **21** | N1457-V332 | 4.6 | D1495-S121* | 2.1 |  |  |  |  |
| **22** | T1443-Y122 | 4.5 | E1462-D336 | 2.1 |  |  |  |  |
| **23** | N1457-D346 | 4.3 | W1496-R214 | 1.9 |  |  |  |  |
| **24** | R1493-A218 | 3.4 | S1468-E312 | 1.7 |  |  |  |  |
| **25** | R1445-E174 | 3.4 | R1493-R216 | 1.6 |  |  |  |  |
| **26** | R1448-N313 | 3.0 | T1464-N313 | 1.3 |  |  |  |  |
| **27** | D1495-R216 | 2.7 | T1464-D126 | 1.0 |  |  |  |  |
| **28** | S1499-E312 | 2.2 |  |  |  |  |  |  |
| **29** | G1456-Q319 | 1.9 |  |  |  |  |  |  |
| **30** | D1495-S121* | 1.7 |  |  |  |  |  |  |
| **31** | N1457-T328 | 1.3 |  |  |  |  |  |  |
| **32** | N1457-T329 | 1.1 |  |  |  |  |  |  |
| **33** | Q1461-N316 | 1.1 |  |  |  |  |  |  |
