# Supplementary figures and images for: High Affinity vs. Native Fibronectin in the Modulation of αvβ3 Integrin Conformational Dynamics: Insights from Computational Analyses and Implications for Molecular Design
Source: PLoS Comput Biol. 2017 Jan 23;13(1):e1005334. doi: 10.1371/journal.pcbi.1005334 (PMC5293283; doi:10.1371/journal.pcbi.1005334)

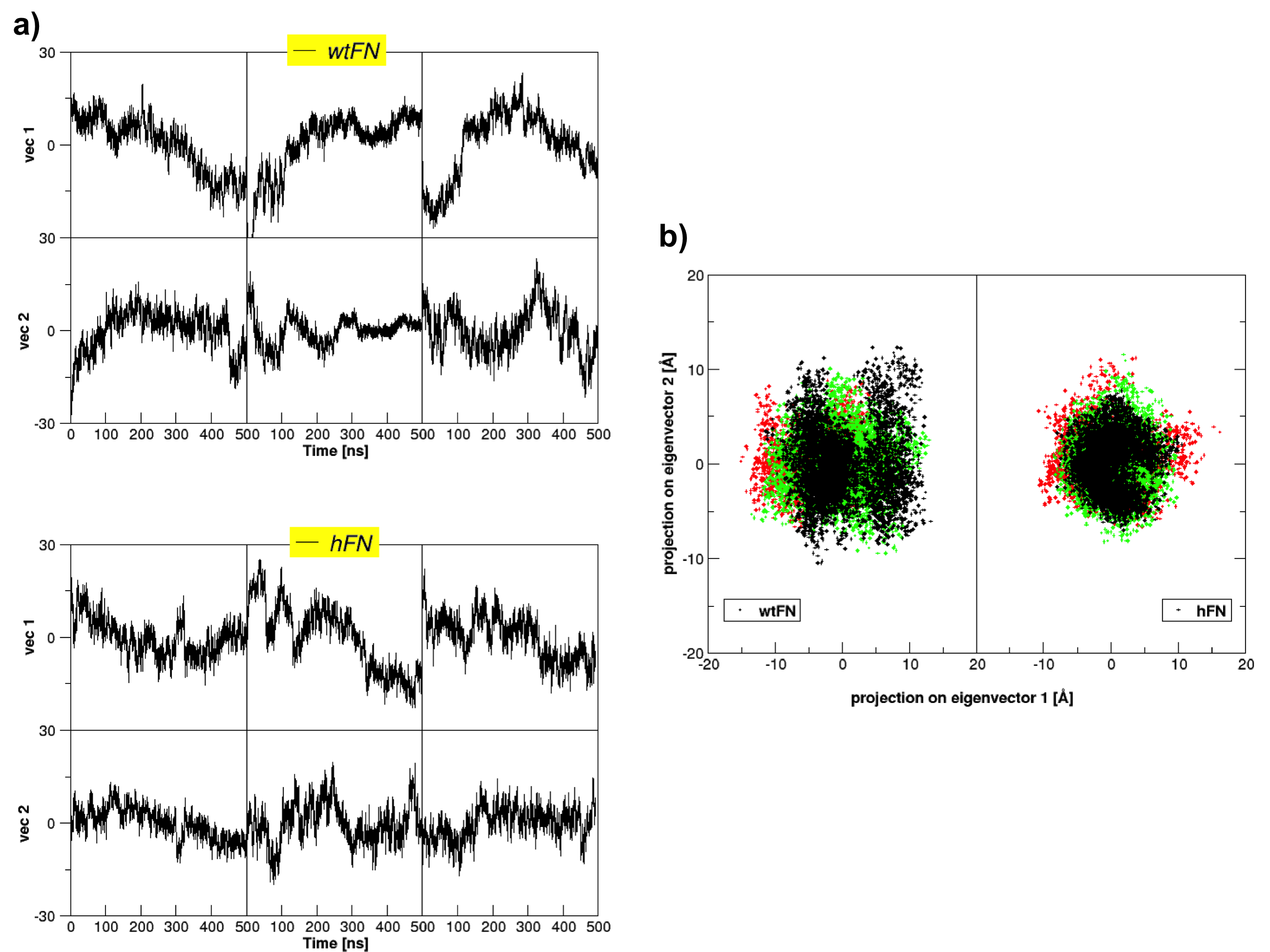

Supplement: S1 Fig — a) Time-dependent projections of wtFN and hFN onto the first two principal modes per replica. b) Projection of wtFN and hFN onto the first two principal modes of the three simulations. Calculations are carried out on C-alpha atoms (979 particles) of αvβ3. Reference structure used for the fitting of either systems is integrin in the wtFN complex (pdb code: 4MMX). The two eigenvectors account for the 55% (wtFN10) and 46% (hFN10) of the total variance of the simulations. (TIF) [file pcbi.1005334.s004.tif]

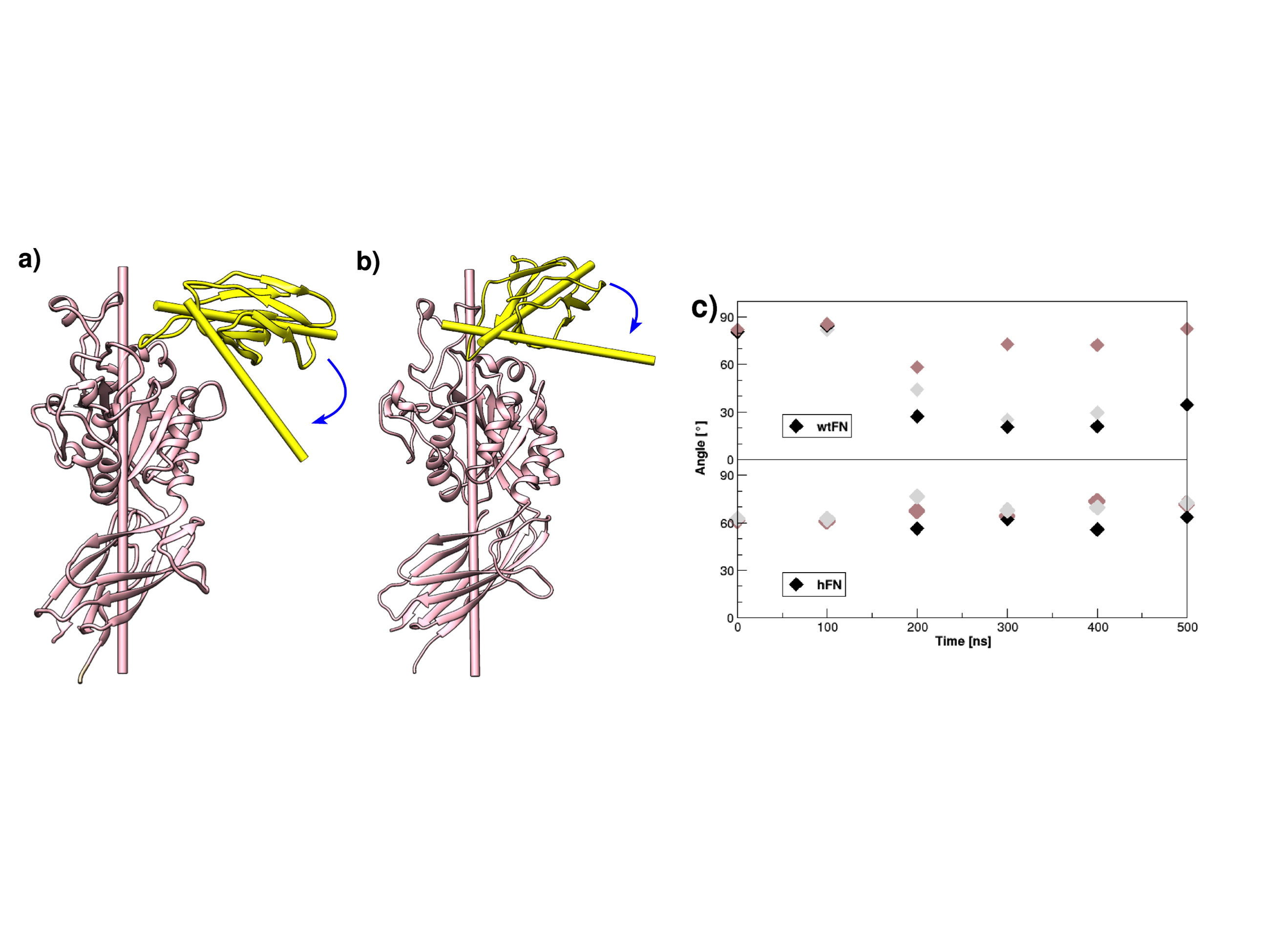

Supplement: S2 Fig — β3 (pink) and Fibronectin (yellow) domains are shown as cartoons in the starting conformation (t = 0) and corresponding principal axes are displayed. Blue arrows indicate the fibronectin torsion angle along the simulation time (replica #1). For clarity, only β3 subunit is shown and the orientation of the two domains in a) and b) displays the maximal value of the distortion. Torsion angles ranges between 21° and 84°(a) and between 56° and 82° (b). c) Time-dependent evolution of the rotational angle described by the principal axes of β3 and FN domains for wtFN10 and hFN10 in the three replicas. See main text. (TIF) [file pcbi.1005334.s005.tif]

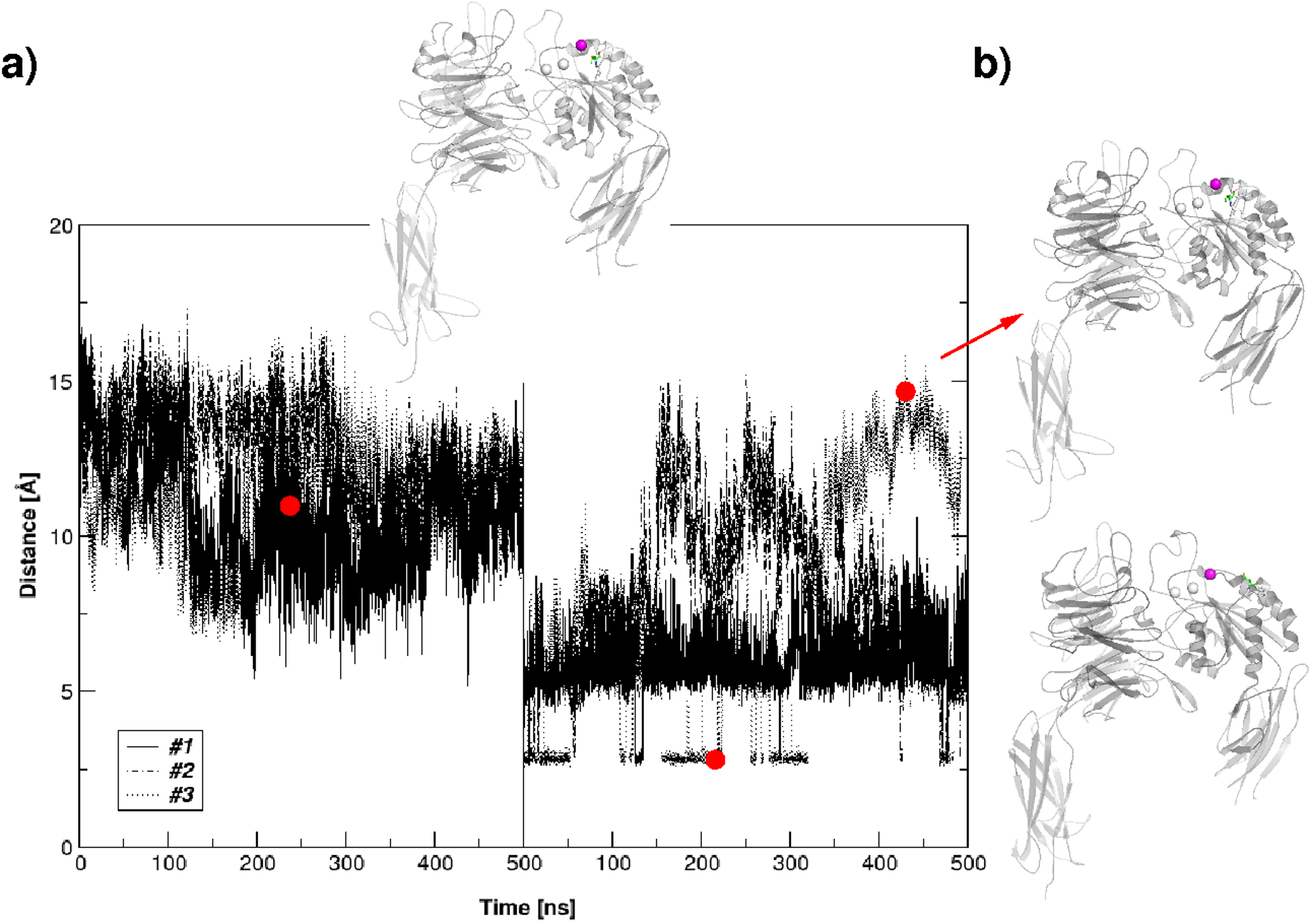

Supplement: S3 Fig — Time evolution series are plotted for each replica (0.5 μs) of wtFN (a) and hFN (b) systems. Integrin conformations corresponding to representative distance values are displayed. For hFN we report structures relative to two extreme distance values. (TIF) [file pcbi.1005334.s006.tif]

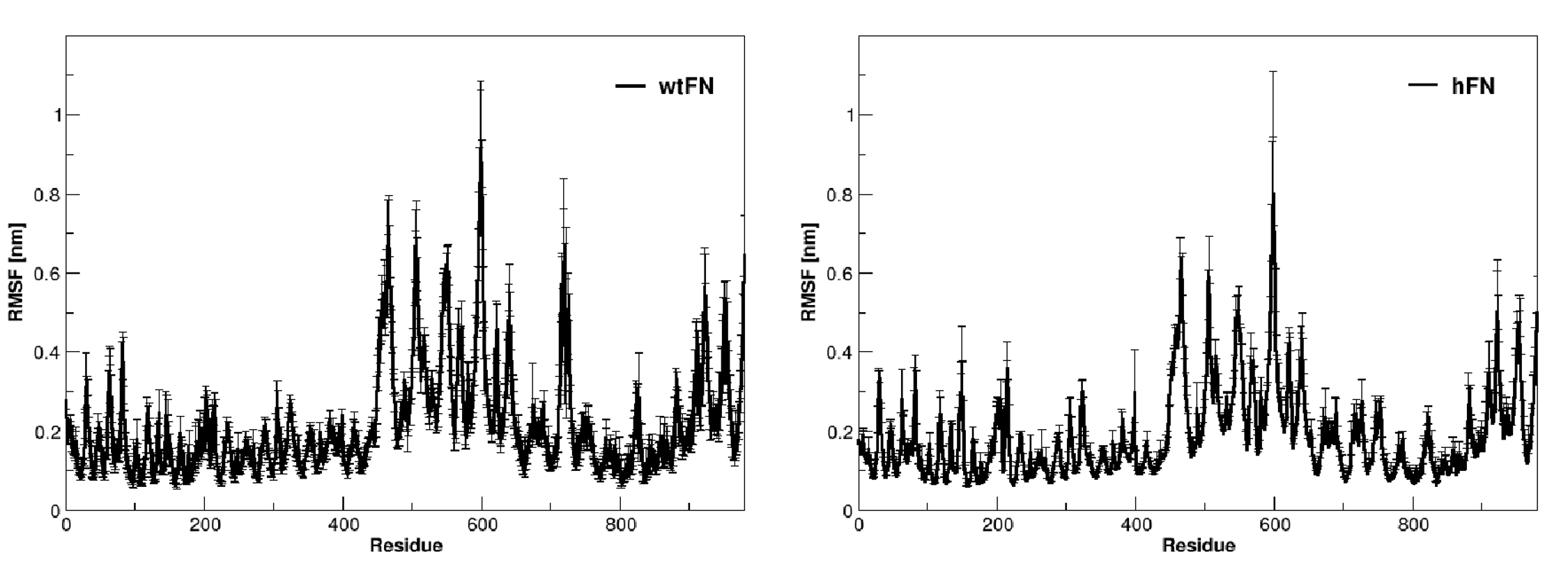

Supplement: S4 Fig — Root mean square fluctuations are plotted per residue (r) and averaged for the 3 replicas along simulation time for wtFN10 (left) and hFN10 (right) systems. Major differences are visible at β3 chain (starting at r = 600). For clarity, fibronectin domain has been omitted. Error bars represent standard deviation. (TIF) [file pcbi.1005334.s007.tif]

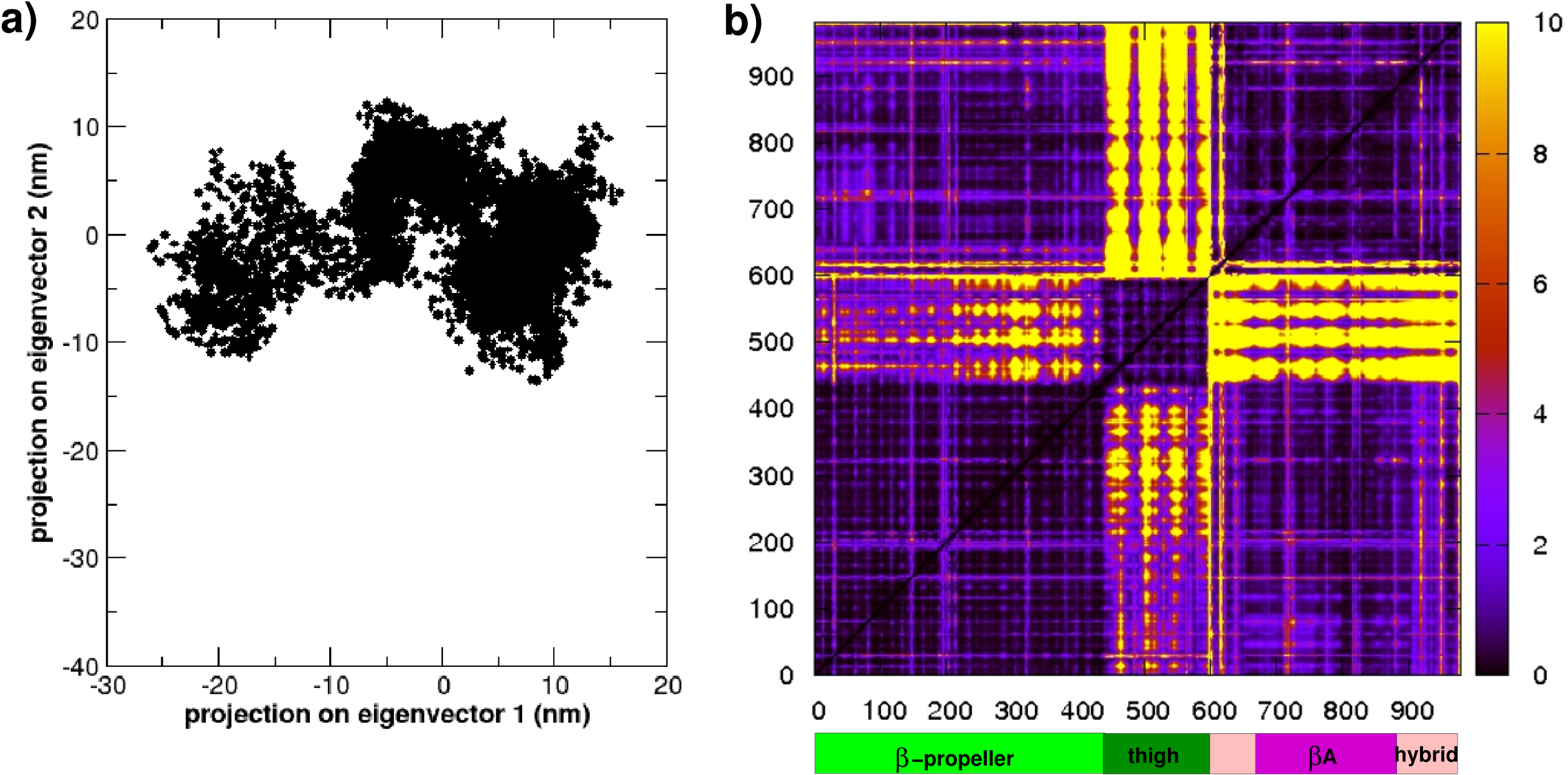

Supplement: S5 Fig — a) ED analysis. Projection of the uncomplexed αvβ3 onto the first two principal modes of the simulation. Calculations are made on the C-alpha atoms. b) DF matrix. Distance Fluctuations are averaged along 500 ns simulation time. Darker spots indicate low inter-residual fluctuation, while lighter striper evidence highly flexible inter-distances. (TIF) [file pcbi.1005334.s008.tif]

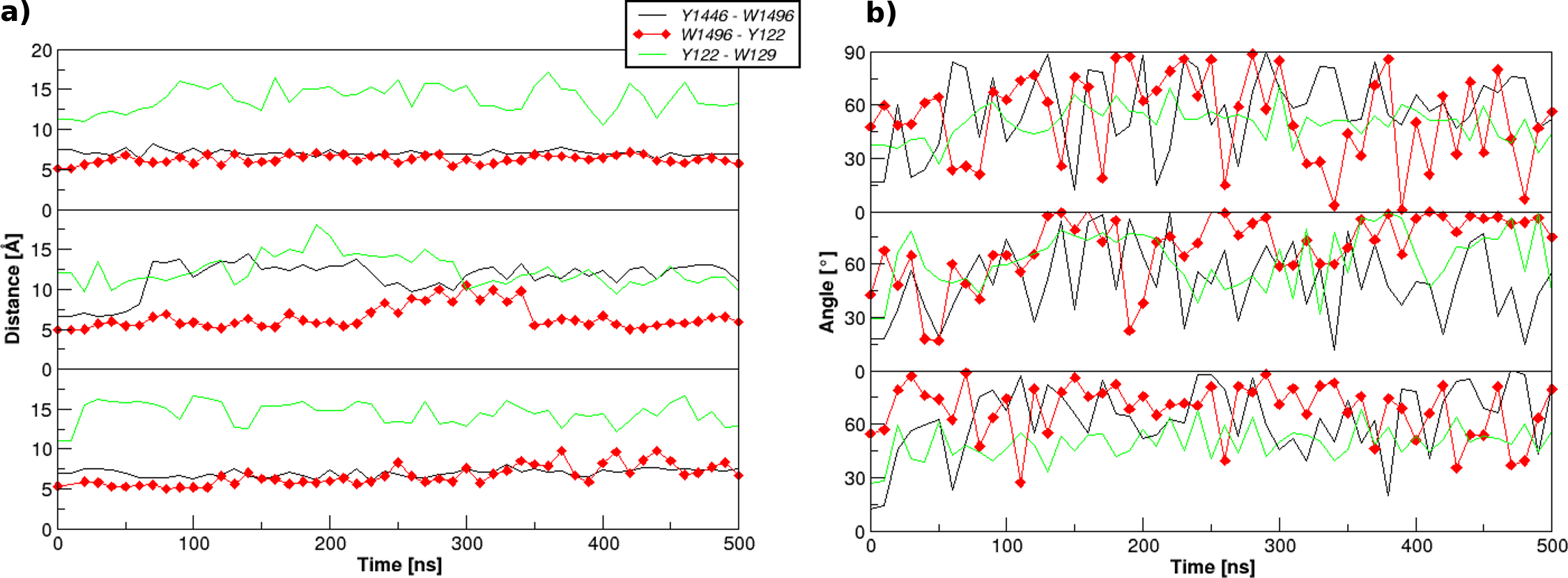

Supplement: S6 Fig — a) Pairwise centroid distances and b) interplanar angle (θ) evolution between Y1446hFN, W1496hFN, Y122β3, W129β3 of the hydrophobic cluster along simulation time (0.5 μs * 3 replicas of hFN10). Red diamonds indicate Y122 β3-W1496hFN packing evolution confirming π- stacking described in ref 3 and discussed along the main text. Note that amino acids are considered paired for centroid distances < 12 Å, while interplanar angle distribution (between 0° and 90°) accounts for stacked and T-shaped conformations. (TIF) [file pcbi.1005334.s009.tif]

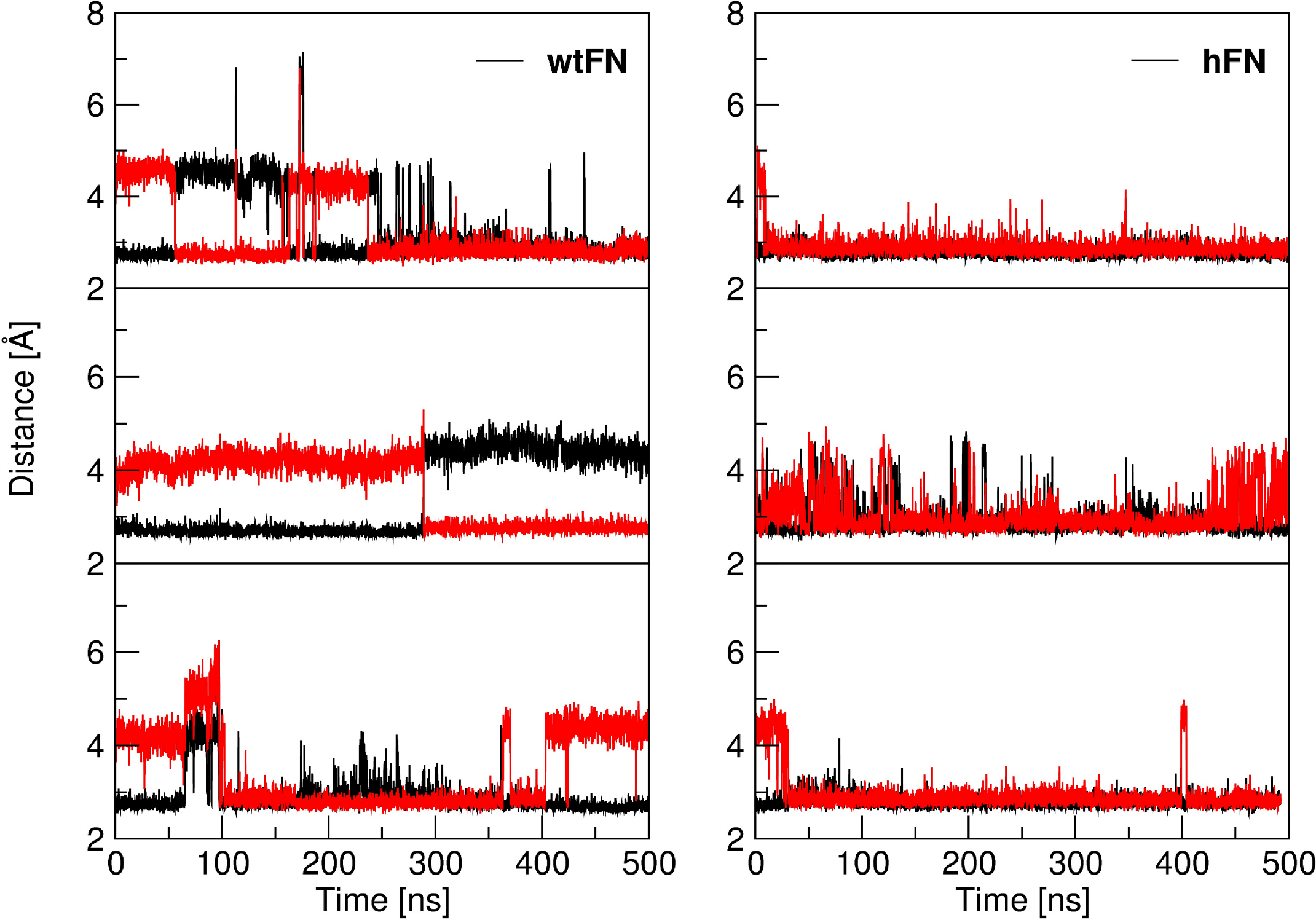

Supplement: S7 Fig — Coordination of Mn2+ at Midas site by carboxylic oxygens- Oδ1 (black) and Oδ2 (red)—from aspartic acid of RGD for individual replica. High flexibility is shown by wtFN10 coordination shell (left panel) compared to the more stable hFN10 system. (TIFF) [file pcbi.1005334.s010.tiff]

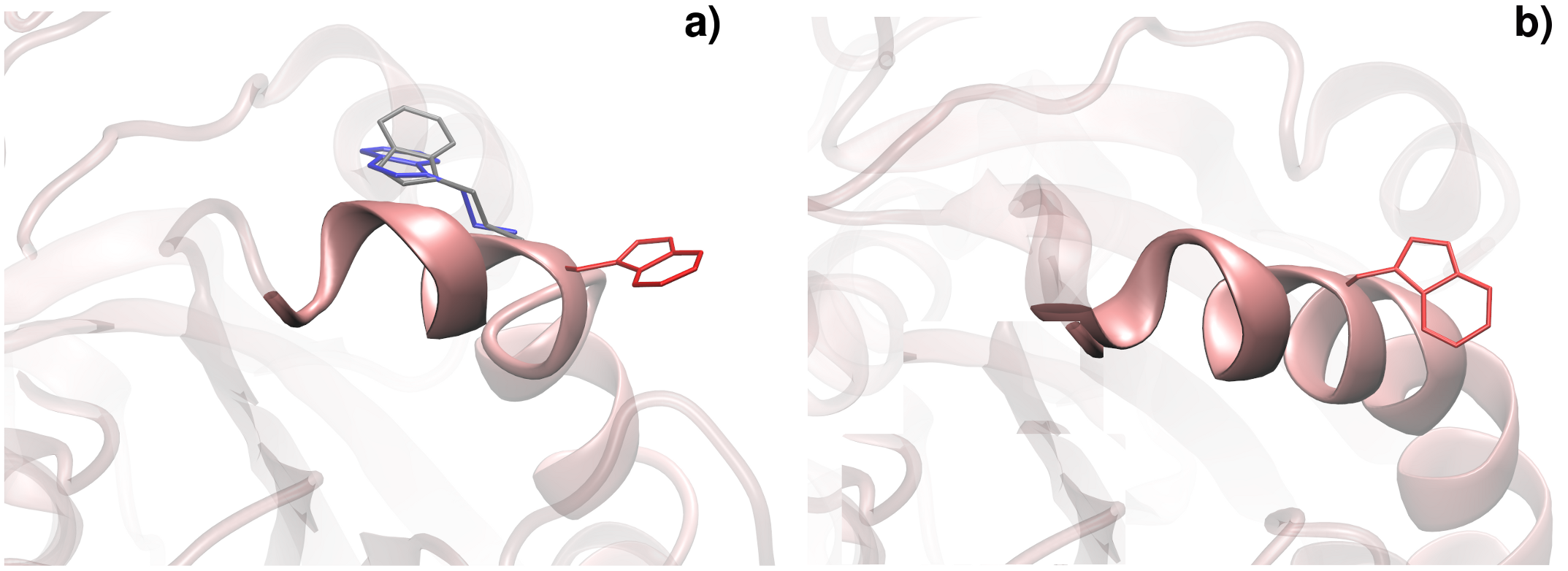

Supplement: S8 Fig — Close-up view of the βA (β3) that directly contacts fibronectin (transparent cartoons). α1 is shown as pink solid cartoons and α1-W129β3 rotamers in sticks. a) Central structure of each cluster for wtFN10 (replica #1) is displayed starting from red (starting frames of the simulation and 0.56% of representativeness) to the gray/blue rotamers (final frames and accounting for the remaining 99.4% of the population). b) Central structure of the unique cluster found for hFN10. Cluster analysis was performed over the region of α1(βA) enclosing W129 (aa. M124-I131) of the β3 chain, using an RMSD cutoff of 0.3 and selecting Gromos method for clustering. [4] 5000 time frames per replica were analyzed. (TIFF) [file pcbi.1005334.s011.tiff]

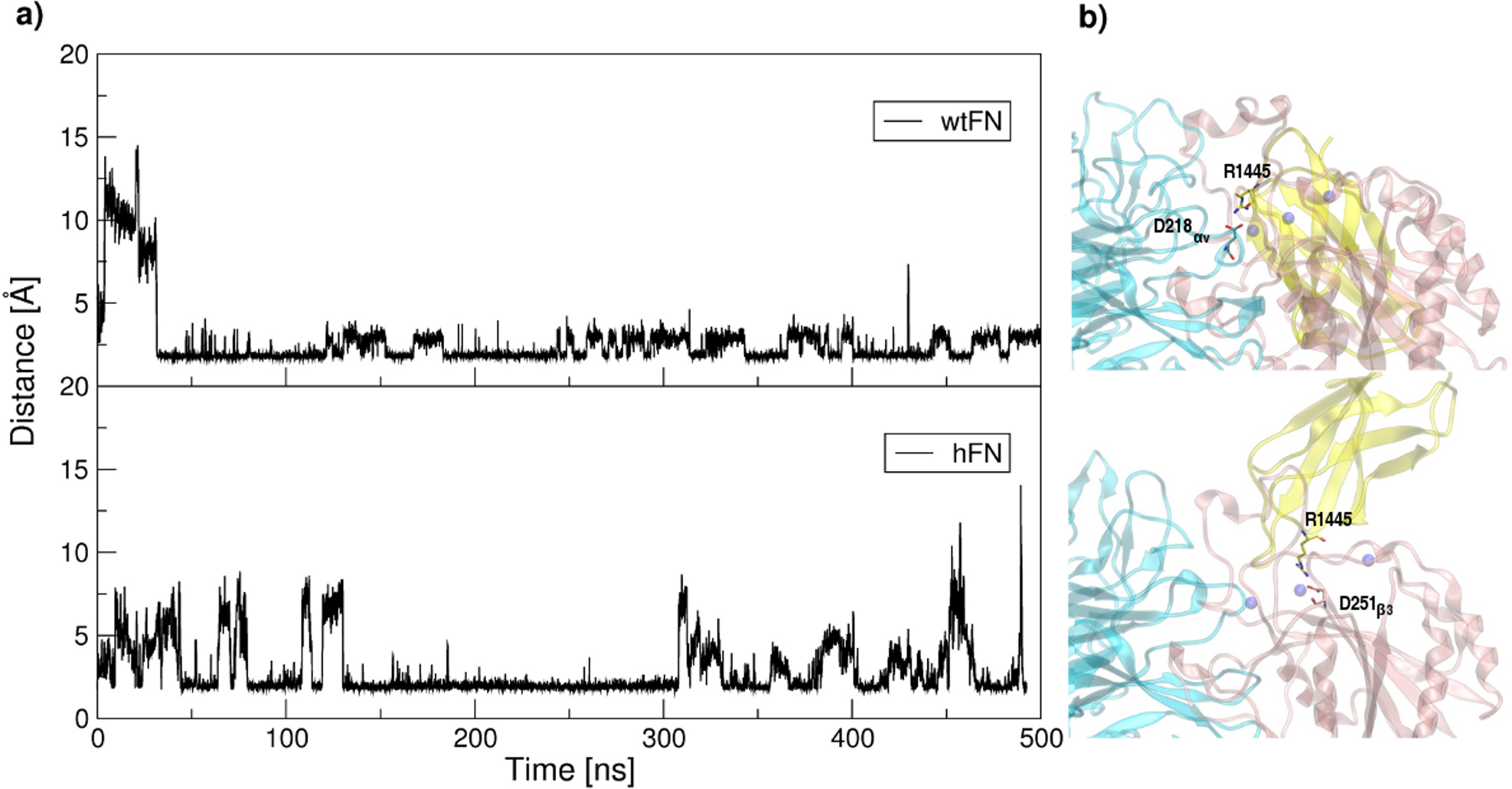

Supplement: S9 Fig — a) Hydrogen bond evolution: wtFN10 D218αv-R1445FN in the top panel, and hFN10 D251β3-R1445FN in the bottom panel. b) Spatial rearrangement of fibronectin in the complex. αvβ3 is in cyan and pink cartoons while FN10 domain is colored yellow. Interacting amino acids are shown in sticks using the same color code of the correspondent subunit. (TIFF) [file pcbi.1005334.s012.tiff]

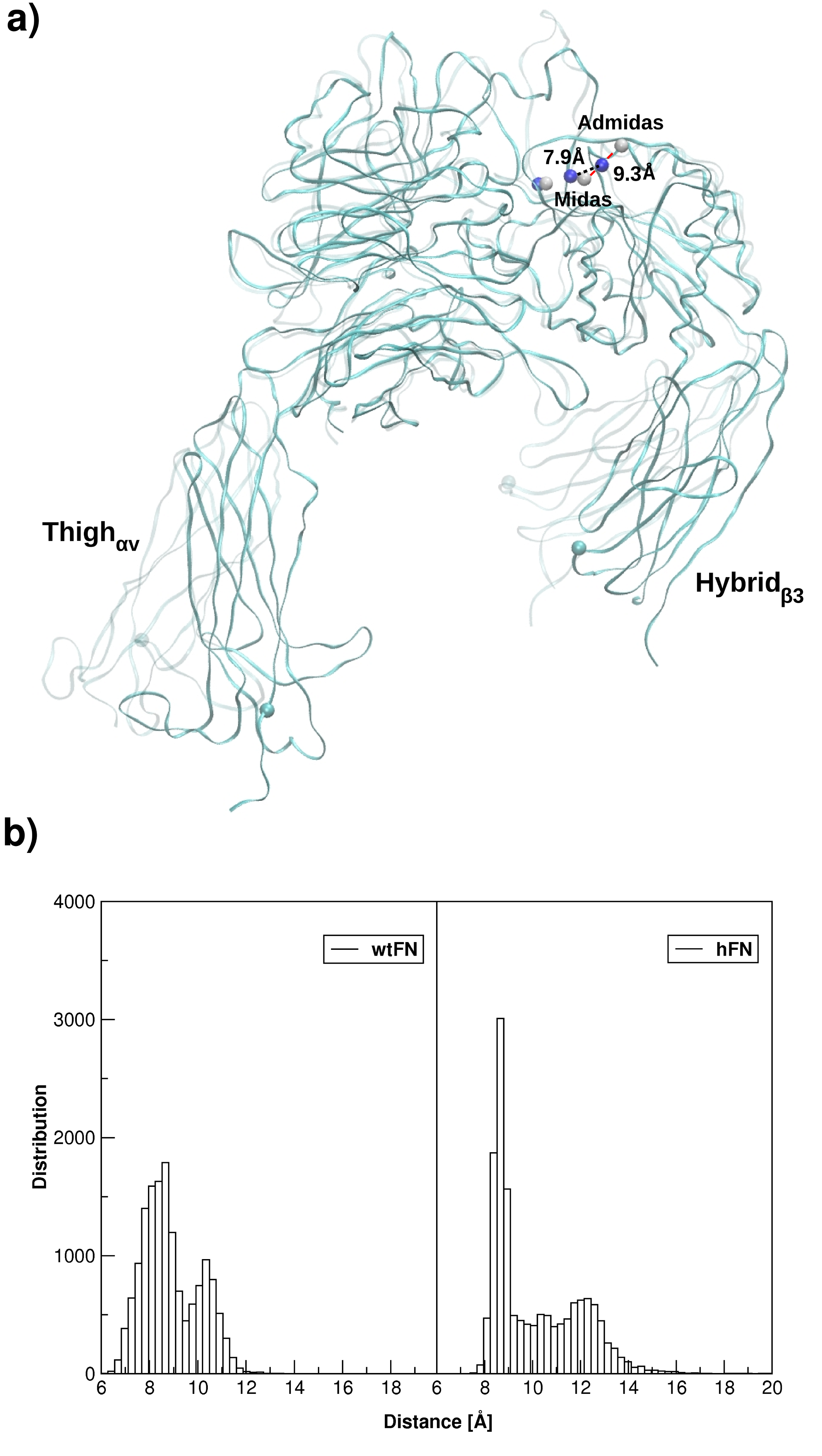

Supplement: S10 Fig — a) Relationship between Mn2+-Mn2+ distances at Midas and Admidas and long-range conformational change. Solid ribbons for αvβ3 and blue spheres for Mn2+ and transparent ribbons and white spheres are used for the two extreme structures of the structural rearrangement. b) Cumulative distribution of Mn2+-Mn2+ interdistances at Midas and Admidas binding sites. Data sets refer to full-length simulation time (1,5 μs) for wtFN10 and hFN10 systems. The main difference between wild type and high affinity FN10 is to be found in the width and height of the bars in the histogram. In this respect it appears that hFN10 is characterized by a dominant peak centered at 8.5 Å, with a queue of less populated bins at higher distances. In the case of wtFN10, the distribution is wider and with bins of comparable populations spanning a larger set of distances. (TIFF) [file pcbi.1005334.s013.tiff]

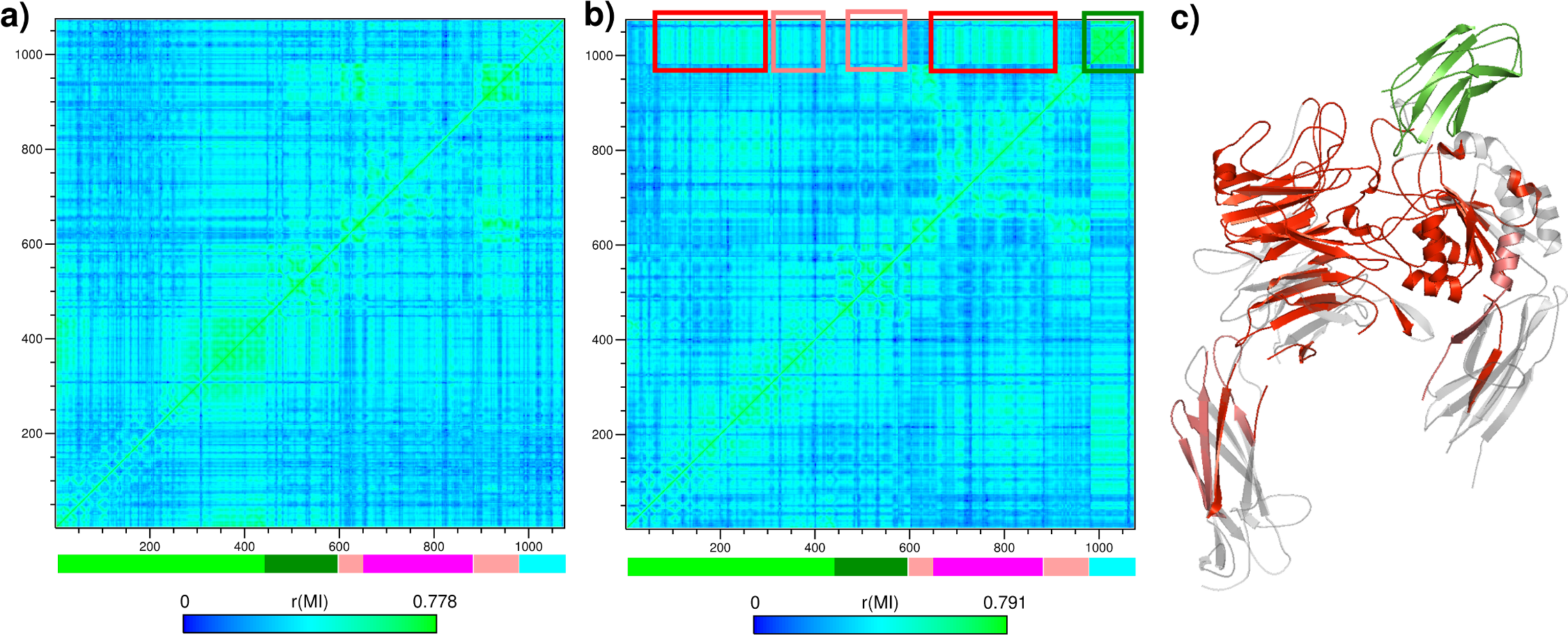

Supplement: S11 Fig — Comparison of generalized correlation coefficient matrices for wtFN10 (a) and hFN10 (b). Data represent average matrices of the three replicas per system (calculated on c-alpha atomic coordinates. c) 3D mapping of the most correlated blocks of hFN10 (color scale of red boxes in b is proportional to correlation). Green box refers to FN domain. The generalized correlation can detect correlated motion regardless of the relative orientation and includes nonlinear contributions. A density estimator nearest-neighbor parameters k = 6 was applied. (TIF) [file pcbi.1005334.s014.tif]
